# Inhibition of the key enzyme of sialic acid biosynthesis by C6-Se modified *N*-acetylmannosamine analogs†
†Electronic supplementary information (ESI) available. See DOI: 10.1039/c5sc04082e


**DOI:** 10.1039/c5sc04082e

**Published:** 2016-02-19

**Authors:** Olaia Nieto-Garcia, Paul R. Wratil, Long D. Nguyen, Verena Böhrsch, Stephan Hinderlich, Werner Reutter, Christian P. R. Hackenberger

**Affiliations:** a Leibniz-Institut für Molekulare Pharmakologie , Robert-Roessle-Strasse 10 , 13125 Berlin , Germany; b Institut für Laboratoriumsmedizin , Klinische Chemie und Pathobiochemie , Charié-Universitätsmedizin Berlin , Arnimalee 22 , 14195 Berlin , Germany . Email: werner.reutter@charite.de; c Beuth Hochschule für Technik Berlin , Department Life Sciences & Technology , Seestrase 64 , 13347 Berlin , Germany . Email: hinderlich@beuth-hochschule.de; d Humboldt Universität zu Berlin , Department Chemie , Brook-Taylor-Strasse 2 , 12489 , Berlin , Germany . Email: hackenbe@fmp-berlin.de

## Abstract

Synthetically accessible C6-analogs of *N*-acetylmannosamine (ManNAc) were tested as potential inhibitors of the bifunctional UDP-*N*-acetylglucosamine-2-epimerase/*N*-acetylmannosamine kinase (GNE/MNK), the key enzyme of sialic acid biosynthesis. Enzymatic experiments revealed that the modification introduced at the C6 saccharide position strongly influences the inhibitory potency. A C6-ManNAc diselenide dimer showed the strongest kinase inhibition in the low μM range among all the substrates tested and successfully reduced cell surface sialylation in Jurkat cells.

## Abstract 2

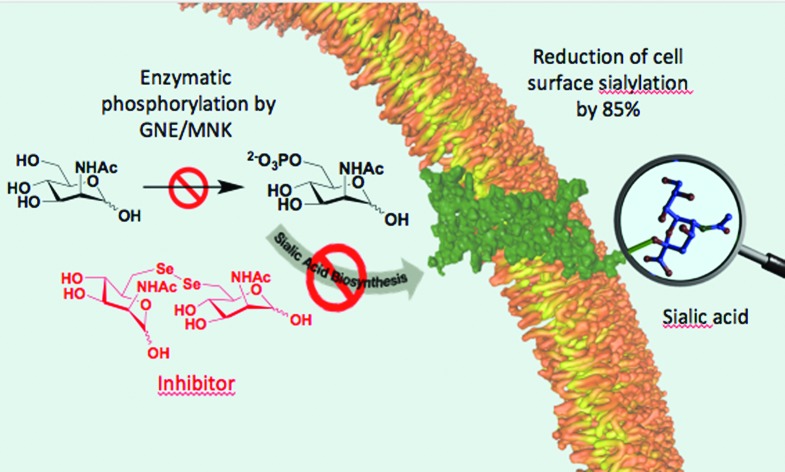
Synthetically accessible C6-analogs of *N*-acetylmannosamine (ManNAc) were tested as potential inhibitors of the bifunctional UDP-*N*-acetylglucosamine-2-epimerase/*N*-acetylmannosamine kinase (GNE/MNK), the key enzyme of sialic acid biosynthesis.

## Introduction

**Scheme 1:**
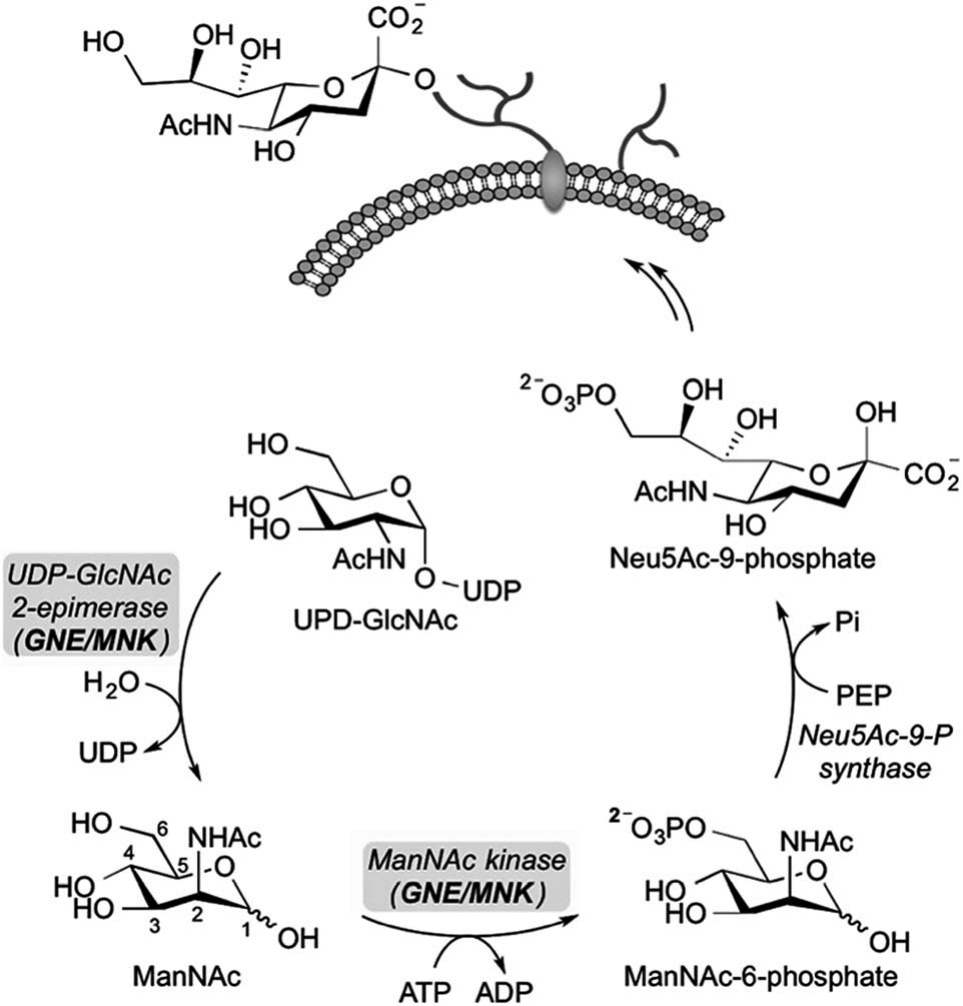
The bifunctional UDP-*N*-acetylglucosamine-2-epimerase/*N*-acetylmannosamine kinase (GNE/MNK) catalyzes the first two steps in the *de novo* synthesis of sialic acids in cells. UDP-GlcNAc is converted by GNE/MNK to ManNAc-6-phosphate, which is then condensed with phosphoenolpyruvate (PEP) to *N*-acetylneuraminic acid-9-phosphate (Neu5Ac-9-P). The latter substrate is further dephosphorylated, activated to CMP-sialic acid and used for the synthesis of sialylated glycans.

Sialic acid is an essential component of the periphery of the glycocalyx. Sialylated glycoconjugates are involved in a broad range of cell functions and biological interactions including recognition of pathogens and immune regulation, among others.^1^–^3^ For instance, it is known that the influenza virus invades host cells through sialic acid recognition.^4^ Many tumor cells show an altered glycosylation pattern with increased expression of sialic acids on their surface.^5^,^6^ Assumingly, this oversialylation may act as a shield to protect these tumor cells from immune recognition.^7^,^8^ Nowadays, the elucidation of the precise role of sialic acids in glycan-mediated cellular interactions is a field of growing interest. For studying cell-surface glycosylation metabolic oligosaccharide-engineering (MOE) is a well-established tool to modify and visualize sialic acids on the cell membrane even in living animals.^9^ By this strategy, unnatural *N*-acetylmannosamine (ManNAc) analogs can be efficiently incorporated into membrane glycoconjugates utilizing the sialic acid biosynthetic pathway, which can be subsequently labeled with bioorthogonal reactions.^9^–^12^ Regarding sialic acid expression, inhibitors of sialyltransferases, a group of enzymes that facilitate the transfer of sialic acid onto glycoconjugates in the Golgi lumen, were reported to efficiently reduce cell surface sialylation *in vitro*^13^,^14^ and *in vivo*.^15^ Another promising approach for the study of sialic acid related biological processes is the development of inhibitors targeting the *de novo* biosynthesis of this sugar. The key enzyme of sialic acid biosynthesis is the bifunctional UDP-*N*-acetylglucosamine-2-epimerase/*N*-acetylmannosamine kinase (GNE/MNK), which consequently serves as an important regulator of cell surface sialylation in mammals.^16^ Specifically, the GNE domain of the enzyme first isomerizes UPD-*N*-acetylglucosamine (UPD-GlcNAc) into ManNAc, which is then phosphorylated at the 6-hydroxy position under consumption of adenosine triphosphate (ATP) by the MNK domain (Scheme 1).

**Fig. 1:**
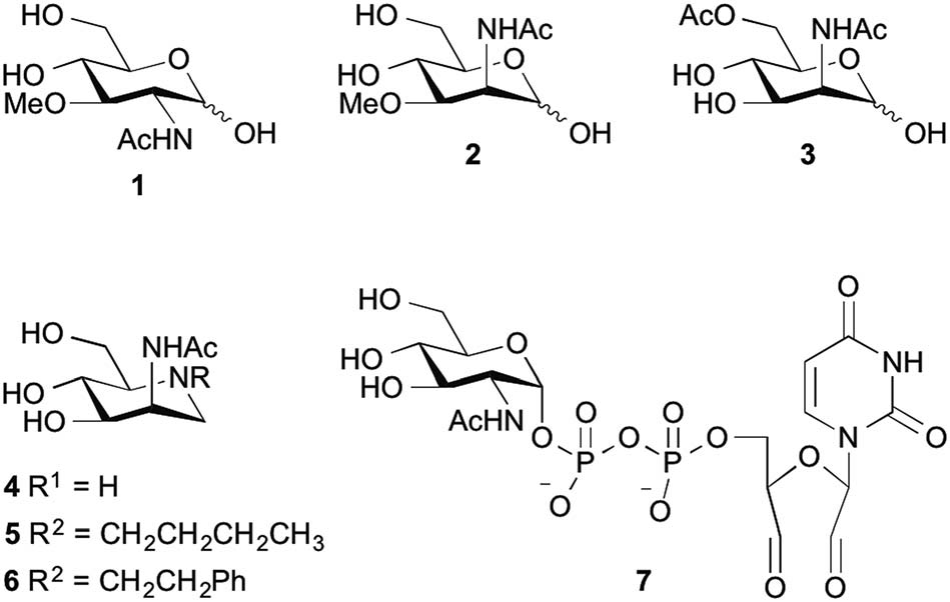
Some unnatural modified monosaccharides reported as inhibitors of GNE/MNK kinase.

To date, known GNE/MNK inhibitors were synthesized based on unnatural monosaccharides, including *N*-acetylglucosamine (GlcNAc), UDP-GlcNAc and ManNAc analogs (Fig. 1).^17^–^23^

Among all reported inhibitors, only 3-*O*-methyl-*N*-acetyl-glucosamine (**1**) and the methylated ManNAc analog **2** were tested in eukaryotic cells. For instance, the methylated GlcNAc analog **1** at 1 mM final concentration reduced the incorporation rate of *N*-GlcNAc and *N*-ManNAc into glycoproteins by 70% in HepG2 cells.^17^ More recently, the methylated ManNAc analog **2** showed reduction of cell surface sialylation to 20% using 500 μM final concentration of the inhibitor in culture medium.^22^ Other reported inhibitors, including 6-*O*-Ac-ManNAc (**3**), iminosugars **4–6** and 2′,3′-dialdehydo-UPD-*N*-acetylglucosamine (**7**) were tested with either the purified ManNAc kinase (**3** at 5 mM final concentration) or UDP-GlcNAc-2-epimerase/MNK kinase (**4–6** at 1 mM; **7** at 300 μM) and showed an inhibition of the corresponding enzyme by 60%, 70% and 93%, respectively.^18^,^20^,^23^ Despite these studies, the need of high inhibitor concentrations for an efficient reduction of cell surface sialylation limits the applicability for further studies, especially for *in vivo* experiments, and points towards the need of novel potent inhibitors for sialic acid *de novo* biosynthesis.

In this manuscript, we report the synthesis and evaluation of sulfur and selenium-based C6-derivatives of *N*-acetylmannosamine as potential inhibitors of the human MNK. We hypothesized that C6-ManNAc derivatives cannot be phosphorylated by MNK, making them competitive inhibitors for this enzyme. Our concept was supported by investigating the crystal structure of human MNK in complex with its natural substrate, ManNAc.^23^ The geometry of the active site revealed that only the hydroxyl moiety at the C6 position of ManNAc is sterically accessible for substrate modifications. For this purpose, we planned to access C6-modified ManNAc analogs, in which larger selenium or sulfur atoms replaced the oxygen at position C6, presumably leading to a better blockage of the binding pocket. Furthermore, we reasoned that these C6-modifications would be promising for inhibitory studies in living cells in contrast to compound **3**, in which the acetylated C6-hydroxyl group is cleaved by endogenous esterases.

## Results and discussion

**Scheme 2:**
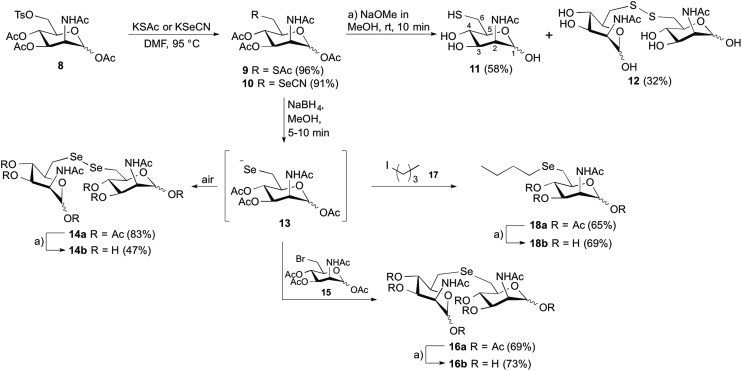
Synthesis of C6-modified *N*-acetylmannosamine analogs.

**Fig. 2:**
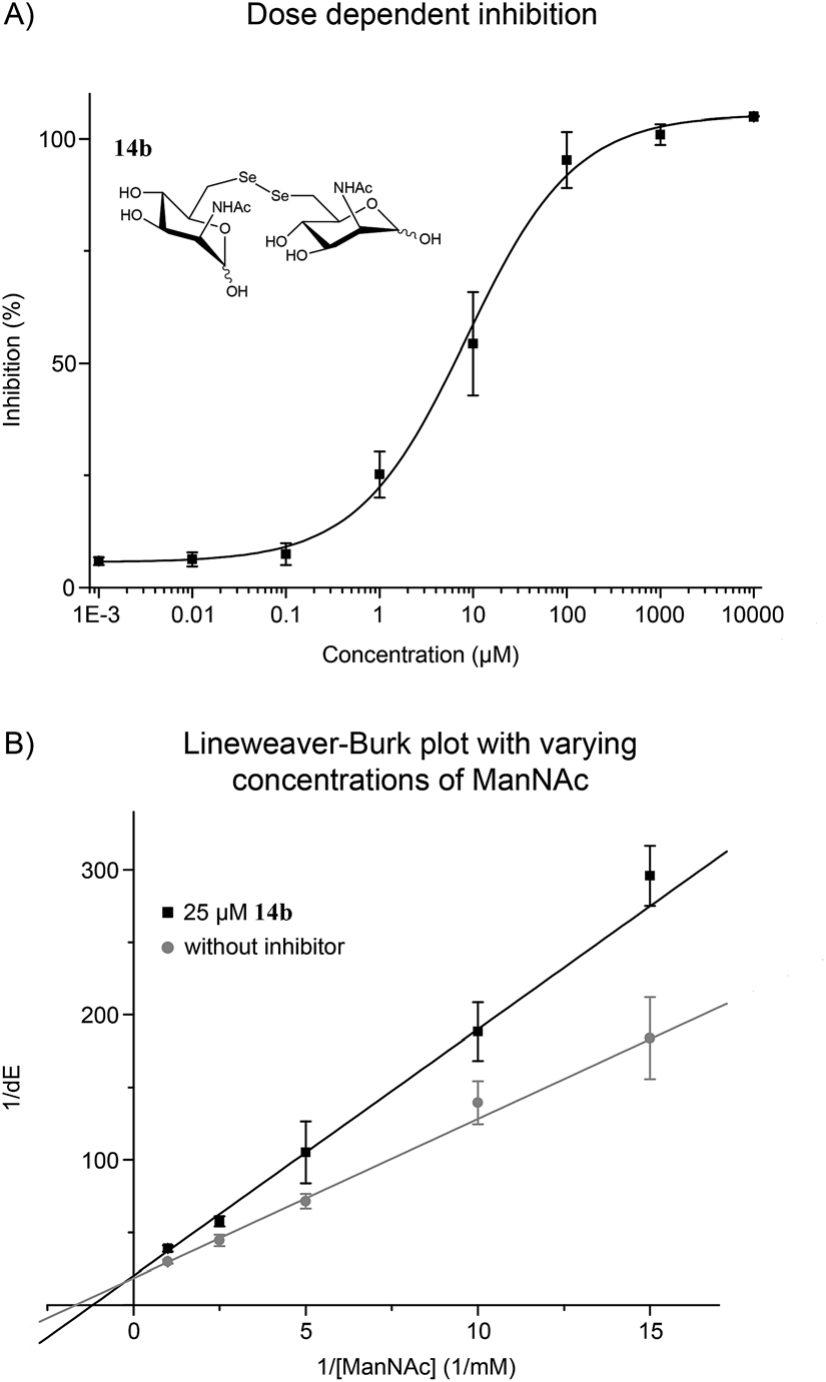
(A) ManNAc diselenide dimer **14b** inhibited the MNK in dose dependent manner. (B) Lineweaver–Burk plot showed a competitive inhibition of **14b** and ManNAc, the natural substrate of MNK. Sigmoidal (A) and linear (B) approximation curves were used to determine IC_50_ and *K*_i_ values. Data shown represent the means ± SEM from five independent experiments.

The synthetic strategy was designed to be versatile and accessible from previously described tosylated ManNAc **8** (Scheme 2),^24^ which can react with appropriate S- or Se-nucleophiles. Considering the S-derivatives, the peracetylated thiol derivative **9** was obtained from **8** by nucleophilic substitution employing potassium thioacetate. In order to obtain water-soluble compounds suitable for enzymatic *in vitro* assays, simultaneous deprotection of *S*- and *O*-acetyl groups was carried out using standard basic conditions; however, to avoid epimerization at position C2 it was crucial to limit the time of basic deprotection with sodium methoxide to 10 min.^25^ We observed the formation of thiol **11** and the disulfide **12** based on the ^1^H-NMR shift of the protons at position C6,^26^ which were both isolated and employed in further enzymatic MNK-assays. To prepare the corresponding C6-Se-ManNAc-analogues, we employed the same electrophilic building block **8** from above. After reaction with nucleophilic potassium selenocyanate as the Se-source, compound **10** was obtained in 91% yield after purification. The chemoselective reduction of selenocyanate in presence of *O*-acetyl groups was ensured by using sodium borohydride, followed by acidification and stirring under inert atmosphere. Although the selenol could be observed after the acidification as a minor peak by UPLC-MS analysis, the high oxidation potential of the selenolate prevented the isolation of the corresponding selenol. Instead, the diselenide dimer **14a** was obtained in 83% yield and analyzed by ^77^Se NMR, displaying the characteristic shift for diselenides at 319–323 ppm. The treatment with sodium methoxide in methanol and subsequent neutralization afforded the unprotected seleno sugar-dimer **14b** in a moderate yield of 47%, most likely due to the cleavage of the labile C–Se bond as previously reported.^27^ With compounds **11**, **12** and **14b** in hand we tested the inhibitory effect in an MNK activity assay. Human MNK was expressed in BL21-CodonPlus (DE3)-RIL *Escherichia coli* (Stragene) and purified according to an established method.^23^ The half maximal inhibitory concentrations (IC_50_) of modified ManNAc analogs for MNK activity have been measured *in vitro* using a coupled optical assay based on consumption of nicotinamide adenine dinucleotide (NADH, details in ESI†). Herein, purified enzyme was incubated with ManNAc (125 μM), the natural substrate for the kinase, ATP and varying concentrations of the previously synthesized C6-modified ManNAc analogs **11**, **12** and **14b**. With an IC_50_ value of 8.5 μM, the diselenide **14b** showed the strongest inhibition of enzyme activity among all tested substances (Table 1, entries 1–3 and Fig. 2A). Encouraged by these results, we also prepared the selenoethers **16b** and **18b**, to further probe the influence of the second selenium atom as well as the second sugar moiety on enzyme activity. Thereby, we took advantage of the nucleophilic properties of the selenolate anion precursor **13** to access the selenoethers. Seleno cyanate **10** was reductively cleaved with sodium borohydride for 5–10 min (monitored by TLC) and the high reactive selenolate anion **13** was caught *in situ* by Ac_3_-6-Br-ManNAc (**15**) or 1-iodopentane (**17**) (Scheme 2). An excess of the electrophiles was used to decrease the formation of the diselenide **14a**. The desired seleno compounds **16a** and **18a** were isolated in 69% and 65%, respectively. The treatment with sodium methoxide delivered unprotected seleno sugars **16b** and **18b** as described for **14b** before. MNK activity studies with selenoethers revealed significantly lower inhibition in comparison to the diselenide **14b** (Table 1, entry 4 and 5).

**Table 1:** Half maximal inhibitory concentrations (IC_50_) of **11**, **12**, **14b**, **16b** and **18b** for MNK inhibition. The data shown represents the means ± SEM obtained in three independent experiments for **11**, **12**, **16b**, **18b** and five independent experiments for **14b**

Entry	Compound	IC_50_ (mM)
1	**11**	>10
2	**12**	4.2 (±0.7)
3	**14b**	8.5 × 10^–3^ (±1.9 × 10^–3^)
4	**16b**	3.0 (±0.7)
5	**18b**	1.9 (±0.5)

The performed MNK activity studies for the sulfur and seleno analogs **11**, **12**, **14b**, **16b** and **18b** indicated that a combination of a diselenide bond together with an additional ManNAc unit is highly beneficial for the enzyme inhibition. In the homodimeric ManNAc derivatives the backbone structure of ManNAc appears twice, giving two potential binding partners for the ManNAc-binding site of MNK. Consequently, this leads to a stochiometrical advantage for dimeric inhibitors, and therefore increases their inhibitory potential compared to monosaccharide ManNAc analogs that have been previously reported as MNK inhibitors.^17^–^23^ Comparing the results of the enzymatic studies with **14b** and **16b**, the length and type of the selenide-linker is apparently of great importance for MNK inhibition. The C–Se–Se–C linker in **14b** is longer, but also could be more flexible than the C–Se–C linker in **16b**. This feature as well as the length and the angle of the C–Se bond could be essential for successful positioning of the inhibitor into the active binding pocket of the MNK.^28^ In addition, different van der Waals interactions and H-bonding properties of selenium and sulfur atoms could play a role for protein binding.^23^ Even though they share a high structural similarity, compound **12** had an approximately 500-fold higher IC_50_ in comparison to **14b**.

With the potent diselenide compound **14b** in hand, we further studied the binding properties of this potent MNK inhibitor, which is to our knowledge the strongest MNK inhibitor reported to date. Diselenide **14b** inhibited MNK activity in a dose dependent manner (Fig. 2A). Additionally, we performed the enzymatic assay in presence of **14b** (25 μM) and different concentrations of ManNAc and confirmed a competitive inhibition of **14b** for ManNAc. The inhibitor constant (*K*_i_) value obtained was 15.7 μM (Fig. 2B). Additionally, we tested if diselenide **14b** influenced the GNE activity of the bifunctional GNE/MNK. Analysis of GNE/MNK in mouse liver cytosol revealed an IC_50_ of **14b** for inhibiting the GNE activity in the range of 200 μM (details in ESI†). This result indicates that the main inhibitory effect of **14b** on sialic acid biosynthesis is due to MNK inhibition, although the GNE activity appears to be influenced by the diselenide **14b**, likely due to an allosteric effect of the binding of the inhibitor to the MNK domain. To evaluate the specificity of the inhibitor towards other sugar kinases, we performed further inhibition studies using human GlcNAc kinase (GNK) and hexokinase (HK) from yeast (details in ESI†). The IC_50_ of **14b** for GNK activity was about 1.7 mM, and higher than 5 mM for HK activity, respectively, indicating that the effects of **14b** on these other sugar kinases are non-specific and negligible.

At this stage, we also wanted to evaluate the stability of the diselenide **14b** in presence of thiol-containing reducing agents like dithiothreitol (DTT) or glutathione (GSH) to address the application of our compounds in living cells. Therefore, the possible reduction of the diselenide bond was monitored by ^77^Se NMR using a solution of 60 mM concentration of **14b** in an aqueous saturated DTT solution at room temperature. Immediately after the addition of **14b** to the DTT solution, a characteristic signal for the selenol compound at –48.1 ppm was observed and a major peak at 294 ppm related to the diselenide. Concretely, the integration of diselenide/selenol peaks gave a 90 : 10 ratio (details in ESI†). Due to the most stable oxidation state, in the time period between 15 min and 5 h, only signals from the diselenide were detected. In the case of saturated GSH solution, using the same procedure as before, only diselenide **14b** was detected after incubation at 60 mM.

**Fig. 3:**
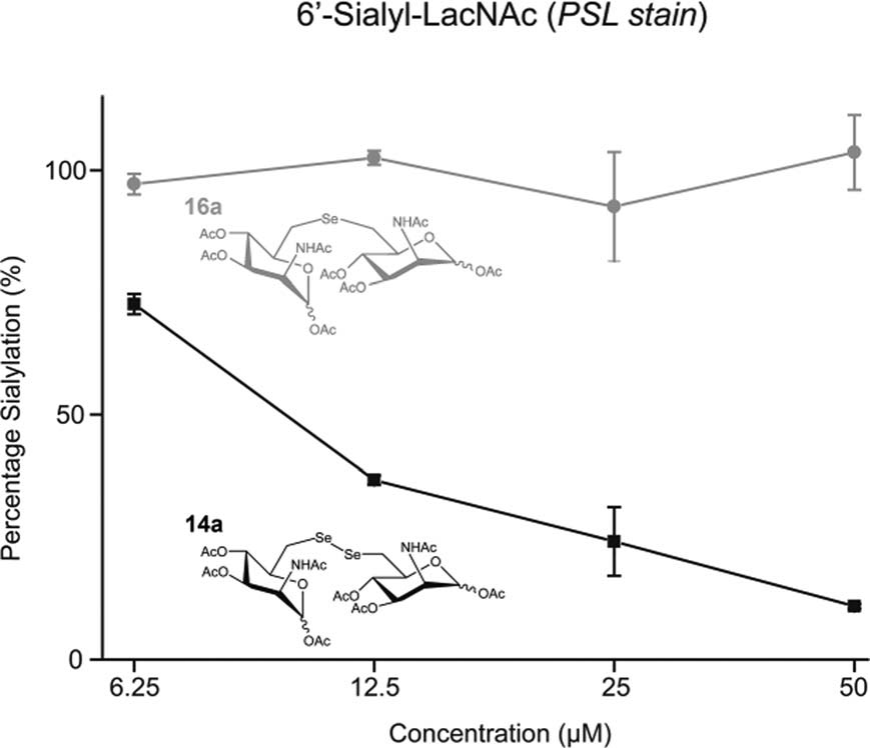
Diselenide **14a** reduced 6′-sialyl-LacNAc expression in Jurkat cells. Selenide **16a** had no visible effect on cell surface sialylation. Data shown represent the means ± SEM obtained in a triplicate.

Encouraged by the strong *in vitro* inhibition of **14b** and its high stability in the presence of reducing agents, we evaluated the capability of this substance to reduce cell surface sialylation. Due to the fact that peracetylated ManNAc derivatives are known to have better membrane permeability,^29^ the corresponding peracetylated diselenide **14a** was used as a prodrug like precursor of **14b** for cell experiments. Additionally, selenide **16a** was chosen as a control because it shares high structural similarity to **14a**, but had only weak effects on the enzyme activity of MNK (Table 1). Jurkat cells were treated for 72 h with different concentrations of peracetylated diselenide **14a** and selenide **16a**. First, the cytotoxicity of **14a** as well as **16a** was evaluated using the AlamarBlue© cell viability assay. Up to a concentration of 50 μM in culture medium, diselenide **14a** exposed negligible cytotoxicity (details in ESI†). However, at a final concentration of 100 μM cell viability was reduced by approximately 70%. Therefore, the following experiments on cell surface sialylation were limited to an inhibitor concentration of 50 μM in culture medium. In the same concentration range, selenide **16a** reduced cell viability by up to 25%. Cell surface sialylation was evaluated by measuring the expression of 6′-sialyl-*N*-acetyllactosamine (6′-sialyl-LacNAc) *via* flow cytometry using fluorescein isothiocyanate-conjugated *Polyporus squamosus* lectin (FITC–PSL).^22^,^30^ Only treatment with diselenide **14a** led to a significant reduction of sialic acid expression in tested cells. At a final concentration of 50 μM in cell culture medium, the 6′-sialyl-LacNAc entity was reduced by approximately 85% (Fig. 3).

As expected, selenide **16a** did not alter cell surface sialylation at tested conditions. Total inhibition of cell surface 6′-sialyl-LacNAc expression was not observed. This is most likely due to uptake and reutilization of sialic acids from the serum supplement and its conversion to CMP-sialic acid independent of GNE/MNK activity.^31^ A slow turnover of sialylated glycoproteins could additionally contribute to this finding.^32^

## Conclusions

In summary, the performed enzymatic assays showed that diselenide **14b** is a potent MNK inhibitor both *in vitro* and *in cellulo* with an IC_50_ value of 8.5 μM, representing the strongest inhibition of all previously reported MNK inhibitors. The homodimeric structure including a diselenide linker introduced at position C6 proved to be highly beneficial for the inhibitory activity of the compound. Finally, we demonstrated that the peracetylated diselenide **14a** was successfully capable of reducing cell surface sialylation in Jurkat cells. With the C6-modified ManNAc analogs presented in this work new insight could be attained into inhibition of the key enzyme of sialic acid biosynthesis, the GNE/MNK. We hope that strong GNE/MNK inhibitors like the described **14a**/**14b**, pave the way for future studies elucidating the effects of GNE/MNK inhibition *in vivo*.

## Supplementary Material

Supplementary information
