# Supplementary material for: Inhibition of the key enzyme of sialic acid biosynthesis by C6-Se modified N-acetylmannosamine analogs
Source: Chem Sci. 2016 Feb 19;7(6):3928–33. doi: 10.1039/c5sc04082e (PMC6013775; doi:10.1039/c5sc04082e)
Supplement: Supplementary file 1 [file SC-007-C5SC04082E-s001.pdf]

Electronic Supplementary Material (ESI) for Chemical Science.
This journal is © The Royal Society of Chemistry 2016

Electronic Supporting Information (ESI)

Inhibition of the key enzyme of sialic acid biosynthesis by C6-Se modified N-acetylmannosamine analogs

*O. Nieto-García,[+,a] P. R. Wratil,[+,b] L. D. Nguyen,[b] Verena Böhrsch,[a] S. Hinderlich, *[,c] W. Reutter,*[,b] and C. P. R. Hackenberger*[a,d]*

[a]Leibniz-Institut für Molekulare Pharmakologie, Robert-Roessle-Strasse 10, 13125 Berlin, Germany

[b]Institut für Laboratoriumsmedizin, klinische Chemie und Pathobiochemie, Charité-Universitätsmedizin Berlin, Arnimalee 22, 14195 Berlin Germany

[c]Beuth Hochschule für Technik Berlin, Department Life Sciences & Technology, Seestrase 64, 13347 Berlin, German

[d]Humboldt Universität zu Berlin, Department Chemie, Brook-Taylor-Strasse 2, 12489, Berlin, Germany

[+]Both authors contributed equally to this work

Email: hinderlich@beuth-hochschule.de, werner.reutter@charite.de or hackenberger@fmp-berlin.de

Contents:

General Remarks	S1
Experimental procedures for **9**, **10**, **11**, **12**, **14a**, **14b**, **15**, **16a**, **16b**, **18a** and **18b**	S2
N-Acetylmannosamine kinase and *N*-acetylglucosamine kinase expression and purification	S7
N-Acetylmannosamine kinase, *N*-acetylglucosamine kinase and yeast hexokinase inhibition experiments	S8
UDP-GlcNAc-2-epimerase activity assays	S9
Cytotoxicity studies	S10
Determination of cell surface sialylation	S10
NMR spectra for new compounds	S13
Evaluation of the stability of the diselenide **14b** in presence of Dithiothreitol (DTT)	S27

General Remarks

Reagents and dry solvents were purchased in the highest available commercial quality and used as supplied. Reactions were monitored by TLC with pre-coated silica gel 60 F254 aluminum plates using UV light as the visualizing agent and by dipping the plate into a solution of *p*-anisaldehyde/H_2SO_4 in EtOH, followed by heating. Flash column chromatography was performed with silica gel (0.035-0.070 mm, 60 Å). Concentrations were carried out in a rotary evaporator. ^{1}H, ^{13}C, dept and ^{77}Se NMR

were recorded using either Bruker Ultrashield 300 MHz spectrometer or Bruker Ultrashield AV 600 MHz, as indicated. Chemical shifts are reported in ppm and coupling constants in Hz; multiplicities are given as follows: s (singlet), br s (broad singlet), br d (broad doublet), d (doublet), dd (doublet of doublets), ddd (doublet of double doublets), t (triplet), and m (multiplet). The composition (*-α,α-*, *-β,β-*, *-α,β-* or *-β,α-*) of the disaccharides **12**, **14a**, **14b**, **16a** and **16b** could not be determined. Therefore, NMR data was given for the single anomers: alpha or beta. Mass spectra were recorded on Agilent 6210 ToF LC/MS system and an Acquity UPLC System (Waters) coupled to an ESI-TOF (electrospray-time of flight) unit LCT Premier (Waters Micromass Technologies). Protein purification was carried out on an Äkta™ purifier system (GE Healthcare). Enzyme activity studies were performed using a Multiskan GO plate reader (Thermo Scientific). For fluorescence-activated cell sorting experiments a FACSCANTO II flow cytometer (BD Biosciences) was used. To determine IC_{50}- and K_i-values, sigmoidal and linear curve approximations were performed using Origin 8.5 Pro (Perkin Elmer).

Experimental procedures

2-Acetamido-1,3,4-tri-O-acetyl-6-S-acetyl-2,6-dideoxy-6-thio-α,β-D-mannopyranose (9)

Potassium thioacetate (341 mg, 2.99 mmol) was added to a solution of **8** (500 mg, 0.9 mmol) in dry DMF (8 mL) under argon. After stirring for 20 h at 95 °C (external bath temp), the solvent was removed *in vacuo*. Chromatography (60% EtOAc/hexane) afforded thiosugar **9** as a foam (388 mg, 96%): R_f (80% EtOAc/hexane) = 0.40; *alpha anomer*: ^{1}H NMR (300 MHz, $CDCl_3$) δ: 5.95 (d, *J* = 1.6 Hz, 1H), 5.84 (d, *J* = 9.2 Hz, 1H), 5.28 (dd, *J* = 10.1, 4.5 Hz, 1H), 5.07 (t, *J* = 10.1 Hz, 1H), 4.59 (ddd, *J* = 9.2, 4.5, 1.6 Hz, 1H), 3.99 (td, *J* = 10.1, 4.8 Hz, 1H), 3.04-3.16 (m, 2H), 2.34 (s, 3H), 2.14 (s, 3H), 2.10 (s, 3H), 2.06 (s, 3H), 1.98 (s, 3H); ^{13}C NMR (75 MHz, $CDCl_3$) δ: 194.6, 170.3, 170.2, 170.1, 168.4, 91.8, 71.3, 68.9, 67.7, 49.5, 30.6, 30.1, 23.5, 21.0 (2C), 20.9; *beta anomer*: ^{1}H NMR (300 MHz, $CDCl_3$) δ: 5.84-5.74 (m, 2H), 5.09-5.02 (m, 2H), 4.78-4.68 (m, 1H), 3.78-3.64 (m, 1H), 3.04-3.16 (m, 2H), 2.34 (s, 3H), 2.14 (s, 3H), 2.10 (s, 3H), 2.06 (s, 3H), 1.98 (s, 3H); ^{13}C NMR (75 MHz, $CDCl_3$) δ: 194.5, 170.8, 170.3, 170.3, 168.5, 90.8, 74.8, 71.5, 67.5, 49.8, 30.6, 30.1, 23.6, 20.9 (3C); HRMS (ESI-TOF, M+H) *m/z*: calcd. for ($C_{16}H_{24}NO_9S$): 406.1172, found: 406.1165.

*2-Acetamido-1,3,4-tri-O-acetyl-6-Se-cyanyl-2,6-dideoxy-α,β-D-mannopyranose (**10**)*

Potassium selenocyanate (190 mg, 1.32 mmol) was added to a solution of **8** (440 mg, 0.88 mmol) in dry DMF (12 mL) under argon. After stirring for 20 h at 95 °C (external bath temp), the solvent was removed *in vacuo*. Chromatography (50% EtOAc/hexane) afforded selenocyanate **10** as a foam (350 mg, 91%): R_f (80% EtOAc/hexane) = 0.38; *alpha-anomer*: ^{1}H NMR (300 MHz, $CDCl_3$) δ: 6.02 (d, *J* = 1.8 Hz, 1H), 5.94 (d, *J* = 9.1 Hz, 1H), 5.35 (dd, *J* = 10.0, 4.5 Hz, 1H), 5.10 (t, *J* = 10.0 Hz, 1H), 4.65 (ddd, *J* = 9.1, 4.5, 1.8 Hz, 1H), 4.13 (ddd, *J* = 10.0, 7.6, 3.1 Hz, 1H), 3.35 (dd, *J* = 12.9, 3.1 Hz, 1H), 3.06 (dd, *J* = 12.9, 7.6 Hz, 1H), 2.19 (s, 3H), 2.10 (s,

3H), 2.06 (s, 3H), 2.01 (s, 3H); ^{13}C NMR (75 MHz, $CDCl_3$) δ: 170.4, 170.3, 170.0, 168.2, 101.6, 91.6, 70.4, 68.9, 68.4, 49.4, 30.6, 23.3, 20.9, 20.8 (2C); ^{77}Se NMR (114 MHz, $CDCl_3$) δ: 176.2; *beta-anomer*: 5.87-5.80 (m, 2H), 5.08-5.01 (m, 2H), 4.78-4.68 (m, 1H), 3.89-3.75 (m, 1H), 3.37-3.30 (m, 1H), 3.14 (dd, *J* = 12.9, 7.6 Hz, 1H), 2.19 (s, 3H), 2.10 (s, 3H), 2.06 (s, 3H), 2.01 (s, 3H); ^{13}C NMR (75 MHz, $CDCl_3$) δ: 170.9, 170.5, 170.2, 168.5, 101.7, 90.5, 73.8, 71.1, 68.7, 49.6, 30.6, 23.5, 20.9, 20.8 (2C); ^{77}Se NMR (114 MHz, $CDCl_3$) δ: 175.2; HRMS (ESI-TOF, M+H) *m/z*: calcd. for ($C_{15}H_{21}N_2O_8Se$): 437.0463, found: 437.0470.

6-6′-Diselenobis-(2-acetamido-1,3,4-tri-O-acetyl-2,6-dideoxy-D-mannopyranose) ***(14a)***

A mixture of selenocyanate **10** (142 mg, 0.33 mmol) and $NaBH_4$ (12 mg, 0.33 mmol) in dry MeOH (6 mL) was stirred for 10 min at rt under argon. The pH of the mixture was adjusted to 5 with 5% aqueous AcOH and the solvent was evaporated *in vacuo*. Chromatography (80% EtOAc/hexane → 10% MeOH/EtOAc) afforded diselenide **14a** (110 mg, 83%) as a white foam: R_f = 0.18 (100% EtOAc); *alpha anomer*: ^{1}H NMR (600 MHz, $CDCl_3$) δ: 6.14-6.10 (m, 1H), 5.99 (d, *J* = 1.8 Hz, 1H), 5.31 (dd, *J* = 9.9, 4.7, 1H), 5.08 (t, *J* = 9.9 Hz, 1H), 4.63 (ddt, *J* = 9.3, 4.7, 1.8 Hz, 1H), 4.01 (ddd, *J* = 9.9, 6.1, 3.4 Hz, 1H), 3.24 (dd, *J* = 13.0, 3.4 Hz, 1H), 3.10-3.04 (m, 1H), 2.18 (s, 3H), 2.07 (s, 3H), 2.05 (s, 3H), 2.00 (s, 3H); ^{13}C NMR (75 MHz, $CDCl_3$) δ: 170.5, 170.2 (2C), 168.7, 91.7, 72.2, 69.1, 68.7, 49.4, 32.9, 23.3, 21.1, 21.0, 20.9; ^{77}Se NMR (114 MHz, $CDCl_3$) δ: 319.1; *beta anomer*: ^{1}H NMR (600 MHz, $CDCl_3$) δ 5.92 (d, *J* = 9.2 Hz, 1H), 5.90 (d, *J* = 1.8 Hz, 1H), 5.31 (dd, *J* = 9.5, 4.4 Hz, 1H), 5.03 (t, *J* = 9.5 Hz, 1H), 4.77 (ddd, *J* = 9.2, 4.4, 1.8 Hz, 1H), 3.84 (ddd, *J* = 9.5, 7.9, 3.5 Hz, 1H), 3.30-3.25 (m, 1H), 3.02 (dd, *J* = 12.9, 7.9 Hz, 1H), 2.16 (s, 3H), 2.10 (s, 3H), 2.06 (s, 3H), 2.00 (s, 3H); ^{13}C NMR (75 MHz, $CDCl_3$) δ: 170.8, 170.3, 170.2, 168.8, 90.5, 74.3, 71.6, 68.9, 49.7, 33.5, 23.5, 21.0, 21.0, 20.9; ^{77}Se NMR (114 MHz, $CDCl_3$) δ: 323.9; HRMS (ESI-TOF, M+H) *m/z*: calcd. for ($C_{28}H_{41}N_2O_{16}Se_2$): 821.0787, found: 821.0782.

2-Acetamido-1,3,4-tri-O-acetyl-6-bromo-2,6-dideoxy-α,β-D-mannopyranose ***(15)***

Litium bromide (424 mg, 4.89 mmol) was added to a solution of **8** (700 mg, 1.40 mmol) in dry DMF (12 mL) at rt under argon. After stirring for 24 h at 85 °C, the solvent was evaporated. Chromatography (50% EtOAc/hexane) gave bromide **15** as a white foam (550 mg, 96%): R_f (70% EtOAc/hexane) = 0.45; *alpha anomer*: ^{1}H NMR (300 MHz, $CDCl_3$) δ: 6.03 (d, *J* = 1.9 Hz, 1H), 5.95 (d, *J* = 9.5 Hz, 1H), 5.32 (dd, *J* = 9.9, 4.4 Hz, 1H), 5.21 (t, *J* = 9.9 Hz, 1H), 4.62 (ddd, *J* = 9.5, 4.4, 1.9 Hz, 1H), 3.99 (ddd, *J* = 9.9, 4.6, 2.9 Hz, 1H), 3.52 (dd, *J* = 11.3, 2.9 Hz, 1H), 3.38 (dd, *J* = 11.3, 4.6 Hz, 1H), 2.15 (s, 3H), 2.07 (s, 3H), 2.04 (s, 3H), 1.99 (s, 3H); ^{13}C NMR (75 MHz, $CDCl_3$) δ: 170.3, 170.2, 169.7, 168.3, 91.7, 70.9, 68.8, 67.9, 49.2, 31.9, 23.4, 20.9 (2C), 20.8; *beta anomer*: ^{1}H NMR (300 MHz, $CDCl_3$) δ: 5.89 (d, *J* = 1.6 Hz, 1H), 5.87-5.80 (m, 1H), 5.28-5.20 (m, 1H), 5.15-5.05 (m, 1H), 4.74 (dd, *J* = 9.8, 4.7 Hz,

1H), 3.79 (ddd, *J* = 8.6, 4.7, 3.1 Hz, 1H), 3.61-3.50 (m, 1H), 3.43-3.36 (m, 1H), 2.15 (s, 3H), 2.10 (s, 3H), 2.07 (s, 3H), 1.99 (s, 3H); ^{13}C NMR (75 MHz, $CDCl_3$) δ: 170.8, 170.3, 169.7, 168.6, 90.4, 73.8, 71.4, 67.5, 49.5, 31.3, 23.5, 21.0, 20.9, 20.8; HRMS (ESI-TOF, M+H) *m/z*: calcd. for ($C_{14}H_{21}BrNO_8$): 410.0451, found: 410.0452.

6-6′-Selenobis-(2-acetamido-1,3,4-tri-O-acetyl-2,6-dideoxy-D-mannopyranose) ***(16a)***

A solution of selenocyanate **10** (150 mg, 0.34 mmol) in dry THF/MeOH (2:1, 6 mL) was treated with $NaBH_4$ (20 mg, 0.52 mmol) at rt under argon. After stirring for 15 min, bromide **15** (633 mg, 1.55 mmol) was added and the mixture stirred at rt for 1.5 h. The pH of the mixture was adjusted to 5 with a 1 M aqueous solution of HCl and the solvent evaporated *in vacuo*. Chromatography (80% → 100% EtOAc/hexane) gave selenoether **16a** (175 mg, 69%) as a white foam: R_f = 0.24 (100% EtOAc); *alpha anomer*: ^{1}H NMR (600 MHz, $CDCl_3$) δ: 6.25-6.13 (m, 1H), 5.93 (d, *J* = 1.8 Hz, 1H), 5.30 (dd, *J* = 10.0, 4.5 Hz, 1H), 5.17 (t, *J* = 10.0 Hz, 1H), 4.62 (ddd, *J* = 9.4, 4.5, 1.9 Hz, 1H), 4.00 (dt, *J* = 9.4, 3.7 Hz, 1H), 2.87 (dd, *J* = 13.3, 3.7 Hz, 1H), 2.76-2.63 (m, 1H), 2.16 (s, 3H), 2.06 (s, 6H), 1.99 (s, 3H); ^{13}C NMR (75 MHz, $CDCl_3$) δ: 170.5, 170.3, 170.2, 168.6, 91.6, 72.5, 68.9, 68.8, 49.1, 26.4, 23.3, 21.0, 20.9 (2C) ^{77}Se NMR (114 MHz, $CDCl_3$) δ: 114.5; *beta anomer***:** ^{1}H NMR (600 MHz, $CDCl_3$) δ: 6.01 (d, *J* = 9.2 Hz, 1H), 5.83 (d, *J* = 1.6 Hz, 1H), 5.12-5.05 (m, 1H), 5.03 (dd, *J* = 9.7, 3.8 Hz, 1H), 4.74 (ddd, *J* = 9.2, 3.8, 1.6 Hz, 1H), 3.79 (ddd, *J* = 9.7, 6.2, 3.7 Hz, 1H), 2.91 (dd, *J* = 13.0, 3.7 Hz, 1H), 2.76-2.63 (m, 1H), 2.16 (s, 3H), 2.09 (s, 3H), 2.08 (s, 3H), 1.97 (s, 3H); ^{13}C NMR (75 MHz, $CDCl_3$) δ: 170.9, 170.6, 170.3, 168.6, 90.5, 75.2, 72.6, 71.5, 49.7, 26.5, 23.4, 20.9 (2C), 20.8; ^{77}Se NMR (114 MHz, $CDCl_3$) δ: 118.4; HRMS (ESI-TOF, M+H) *m/z*: calcd. for ($C_{28}H_{41}N_2O_{16}Se$): 741.1621, found: 741.1632.

2-Acetamido-1,3,4-tri-O-acetyl-6-Se-butyl-2,6-dideoxy-6-seleno-α,β-D-mannopyranose ***(18a)***

A solution of **10** (80 mg, 0.18 mmol) in dry THF/MeOH (2:1, 3 mL) was treated with $NaBH_4$ (10 mg, 0.28 mmol) at rt under argon. After stirring for 15 min, 1-iodopentane (**17**) (63 μL, 0.55 mmol) was added and the mixture stirred at rt for 1 h. The pH of the mixture was adjusted to 5 with a 1 M aqueous solution of HCl and the solvent evaporated *in vacuo*. Chromatography (50% EtOAc/hexane) gave selenide **18a** (55 mg, 65%) as a white foam: R_f = 0.44 (60% EtOAc/hexane); *alpha anomer*: ^{1}H NMR (600 MHz, $CDCl_3$) δ: 5.97 (d, *J* = 1.6 Hz, 1H), 5.96-5.90 (m, 1H), 5.32 (dd, *J* = 10.5, 3.4 Hz, 1H), 5.22 (t, *J* = 10.5 Hz, 1H), 4.67-4.55 (m, 1H), 4.06-3.95 (m, 1H), 2.86-2.74 (m, 1H), 2.69-2.59 (m, 3H), 2.15 (s, 3H), 2.05 (s, 6H), 1.99 (s, 3H), 1.60 (p, *J* = 7.4 Hz, 2H), 1.38 (h, *J* = 8.1, 7.4 Hz, 2H), 0.90 (t, *J* = 7.4 Hz, 3H); ^{13}C NMR (75 MHz, $CDCl_3$) δ: 170.3 (2C), 170.1, 168.4, 91.6, 72.3, 69.0, 68.8, 49.1, 32.7, 32.6, 25.9, 23.4, 23.0, 21.0, 20.9 (2C), 13.7; ^{77}Se NMR (114 MHz, $CDCl_3$) δ: 131.3; *beta anomer***:** ^{1}H NMR (600 MHz, $CDCl_3$) δ: 5.91 (d, *J* = 9.2 Hz, 1H), 5.83 (d, *J* = 1.4 Hz, 1H), 5.17 (t, *J* = 10.0 Hz, 1H), 5.02 (dd, *J* = 10.0, 4.0 Hz, 1H), 4.73 (ddd, *J* = 9.2, 4.0, 1.4 Hz, 1H), 3.81- 3.75 (m, 1H), 2.86-2.74 (m, 1H), 2.69-2.59 (m, 3H), 2.15 (s, 3H),

2.05 (s, 6H), 1.99 (s, 3H), 1.60 (p, *J* = 7.4 Hz, 2H), 1.38 (h, *J* = 8.1, 7.4 Hz, 2H), 0.90 (t, *J* = 7.4 Hz, 3H); ^{13}C NMR (75 MHz, $CDCl_3$) δ: 170.7, 170.3, 170.0, 168.5, 90.4, 75.3, 71.6, 68.7, 49.7, 32.7, 32.6, 25.7, 23.4, 23.0, 21.0, 20.9 (2C), 13.7; ^{77}Se NMR (114 MHz, $CDCl_3$) δ: 134.4; HRMS (ESI-TOF, M+H) *m/z*: calcd. for ($C_{18}H_{30}NO_8Se$): 468.1137, found: 468.1140.

O-Acetyl deprotection conditions*:* The corresponding peracetalyted precursors **9**, **14a**, **16a** and **18a** were dissolved in MeOH and treated with NaOMe (1 equiv., 5.4 M in MeOH) for 10 min at rt. The pH of the mixture was adjusted to 5 with a 1 M aqueous solution of HCl, the solvent was evaporated and a final silica gel column chromatography (under the elution conditions specified) gave the desired unprotected sugars.

*2-Acetamido-6-S-acetyl-2,6-dideoxy-6-thio-α,β-D-mannopyranose (**11**)*

Thiol **11** [from **9** (150 mg, 0.13 mmol), column eluent (100% EtOAc → 10% MeOH/EtOAc → 20% MeOH/EtOAc), 50 mg, 58%, white solid, R_f (20% MeOH/EtOAc) = 0.5]; *alpha anomer*: ^{1}H NMR (300 MHz, D_2O) δ: 5.10 (s, 1H), 4.28 (d, *J* = 4.9 Hz, 1H), 4.02 (dd, *J* = 9.8, 4.9 Hz, 1H), 3.88 (ddd, *J* = 9.8, 7.3, 2.8 Hz, 1H), 3.58 (t, *J* = 9.8 Hz, 1H), 3.07-2.92 (m, 1H), 2.77-2.67 (m, 1H), 2.03 (s, 3H); ^{13}C NMR (75 MHz, D_2O) δ: 174.7, 92.9, 72.2, 69.2, 68.4, 53.2, 25.0, 21.8; *beta anomer*: ^{1}H NMR (300 MHz, D_2O) δ: 5.02 (d, *J* = 1.7 Hz, 1H), 4.44 (d, *J* = 4.5 Hz, 1H), 3.80 (dd, *J* = 9.2, 4.5 Hz, 1H), 3.50-3.39 (m, 2H), 3.07-2.92 (m, 1H), 2.77-2.67 (m, 1H), 2.07 (s, 3H); ^{13}C NMR (75 MHz, D_2O) δ: 175.6, 92.9, 76.6, 71.6, 69.0, 53.9, 24.9, 21.9; HRMS (ESI-TOF, M+1) *m/z*: calcd. for ($C_8H_{16}NO_5S$): 238.0749, found: 238.0755.

*6-6′-Dithiobis-(2-acetamido-2-deoxy-D-mannopyranose) (**12**)*

Disulfide **12** [from **9** (150 mg, 0.13 mmol), column eluent (100% EtOAc → 10% MeOH/EtOAc → 20% MeOH/EtOAc), 25 mg, 28%, white solid, R_f (20% MeOH/EtOAc) = 0.14]; *alpha anomer*: ^{1}H NMR (600 MHz, D_2O) δ: 5.15 (d, *J* = 1.5 Hz, 1H), 4.35 (dd, *J* = 4.7, 1.5 Hz, 1H), 4.20-4.11 (m, 1H), 4.12-4.02 (m, 1H), 3.40-3.33 (m, 1H), 2.99-2.87 (m, 2H), 2.09 (s, 3H). ^{13}C NMR (151 MHz, D_2O) δ: 174.8, 93.0, 71.8, 70.0, 68.6, 53.4, 39.9, 22.0; *beta anomer*: ^{1}H NMR (600 MHz, D_2O) δ: 5.07 (d, *J* = 1.5 Hz, 1H), 4.52-4.48 (m, 1H), 3.89-3.82 (m, 1H), 3.68-3.58 (m, 1H), 3.49 (t, *J* = 9.7 Hz, 1H), 2.99-2.87 (m, 2H), 2.13 (s, 3H); ^{13}C NMR (151 MHz, D_2O) δ: 175.7, 93.1, 74.5, 70.2, 69.8, 54.1, 39.7, 22.1; HRMS (ESI-TOF, M+H) *m/z*: calcd. for ($C_{16}H_{29}N_2O_{10}S_2$): 473.1264, found: 473.1269.

*6-6′-Diselenobis-(2-acetamido-2-deoxy-D-mannopyranose) (**14b**)*

Diselenide **14b** [from **14a** (110 mg, 0.13 mmol), column eluent (100% EtOAc → 20% MeOH/EtOAc), 36 mg, 47%, white solid, R_f (20% MeOH/EtOAc) = 0.14]; *alpha anomer*: ^{1}H NMR (600 MHz, D_2O) δ: 5.14 (s, 1H), 4.39-4.28 (m, 1H), 4.12-4.04 (m, 1H), 3.65-3.51 (m, 2H), 3.49-3.40 (m, 1H), 3.15-3.05 (m, 1H), 2.09 (s, 3H);

^{13}C NMR (151 MHz, D_2O) δ 174.3, 92.5, 71.3, 70.2, 68.0, 52.9, 30.8, 21.5; ^{77}Se NMR (114 MHz, D_2O) δ: 282.8, 280.3; *beta anomer*: ^{1}H NMR (600 MHz, D_2O) δ: 5.06 (s, 1H), 4.52-4.41 (m, 1H), 3.89-3.83 (m, 1H), 3.65-3.51 (m, 2H), 3.49-3.40 (m, 1H), 3.15-3.05 (m, 1H) 2.13 (s, 3H); ^{13}C NMR (151 MHz, D_2O) δ 175.2, 92.6, 71.1, 70.1, 69.9, 53.7, 30.6, 21.7; ^{77}Se NMR (114 MHz, D_2O) δ: 288.2, 285.5; HRMS (ESI-TOF, M+H) *m/z*: calcd. for ($C_{16}H_{29}N_2O_{10}Se_2$): 569.0153, found: 569.0155.

*6-6´-Selenobis-(2-acetamido-2,6-dideoxy-D-mannopyranose) (**16b**)*

Selenoether **16b** [from **16a** (125 mg, 0.17 mmol), column eluent (100% EtOAc → 10% MeOH/EtOAc), 60 mg, 73%, oil, R_f (10% MeOH/EtOAc) = 0.12]; *alpha anomer*: ^{1}H NMR (600 MHz, D_2O) δ: 5.13 (d, *J* = 1.5 Hz, 1H), 4.33 (d, *J* = 4.8 Hz, 1H), 4.07 (dd, *J* = 9.9, 4.8 Hz, 1H), 4.05-3.98 (m, 1H), 3.58 (t, *J* = 9.9 Hz, 1H), 3.25-3.17 (m, 1H), 2.94-2.84 (m, 1H), 2.09 (s, 3H); ^{13}C NMR (75 MHz, D_2O) δ: 174.7, 92.9, 71.5, 70.4, 68.4, 53.2, 25.4, 21.8; ^{77}Se NMR (114 MHz, D_2O) δ: 98.1; *beta anomer*: ^{1}H NMR (600 MHz, D_2O) δ: 5.06 (d, *J* = 1.8 Hz, 1H), 4.49 (dd, *J* = 4.5, 1.8 Hz, 1H), 4.05-3.99 (m, 1H), 3.84 (dd, *J* = 9.8, 4.5 Hz, 1H), 3.47 (t, *J* = 9.8 Hz, 1H), 3.25-3.17 (m, 1H), 2.94-2.84 (m, 1H), 2.09 (s, 3H); ^{13}C NMR (75 MHz, D_2O) δ: 175.5, 92.8, 76.2, 71.4, 70.7, 53.9, 25.7, 22.0; ^{77}Se NMR (114 MHz, D_2O) δ: 91.9; HRMS (ESI-TOF, M+H) *m/z*: calcd. for ($C_{16}H_{29}N_2O_{10}Se$): 489.0987, found: 489.0987.

*2-Acetamido-6-Se-butyl-2,6-dideoxy-6-seleno-α,β-D-mannopyranose (**18b**)*

Selenoether **18b** [from **18a** (180 mg, 0.38 mmol), column eluent (100% EtOAc → 10% MeOH/EtOAc) , 90 mg, 69%, oil, R_f (10% MeOH/EtOAc) = 0.40]; *alpha anomer*: ^{1}H NMR (600 MHz, D_2O) δ : 5.12 (d, *J* = 1.5 Hz, 1H), 4.32 (dd, *J* = 4.8, 1.5 Hz, 1H), 4.05 (dd, *J* = 9.8, 4.8 Hz, 1H), 3.98 (td, *J* = 9.8, 2.8 Hz, 1H), 3.55 (t, *J* = 9.8 Hz, 1H), 3.15-3.08 (m, 1H), 2.81 (ddd, *J* = 13.2, 8.3, 6.9 Hz, 1H), 2.78-2.65 (m, 2H), 2.08 (s, 3H), 1.70 (p, *J* = 7.4 Hz, 2H), 1.42 (h, *J* = 7.4 Hz, 2H), 0.92 (t, *J* = 7.4 Hz, 3H); ^{13}C NMR (151 MHz, D_2O) δ: 174.3, 92.5, 71.1, 70.4, 68.0, 52.9, 31.4, 24.1, 21.8, 21.6, 21.5, 12.3; ^{77}Se NMR (114 MHz, D_2O) δ: 110.5; *beta anomer*: ^{1}H NMR (600 MHz, D_2O) δ: 5.04 (d, *J* = 1.5 Hz, 1H), 4.49-4.42 (m, 1H), 3.95-3.90 (m, 1H), 3.83 (dd, *J* = 9.7, 4.8 Hz, 1H), 3.54-3.50 (m, 1H), 3.15-3.08 (m, 1H), 2.81 (ddd, *J* = 13.2, 8.3, 6.9 Hz, 1H), 2.78-2.65 (m, 2H), 2.08 (s, 3H), 1.70 (p, *J* = 7.4 Hz, 2H), 1.42 (h, *J* = 7.4 Hz, 2H), 0.92 (t, *J* = 7.4 Hz, 3H); ^{13}C NMR (151 MHz, D_2O) δ: 175.2, 92.5, 75.8, 71.3, 70.1, 53.6, 31.4, 24.3, 21.8, 21.6, 21.5, 12.3; ^{77}Se NMR (114 MHz, D_2O) δ: 115.9; HRMS (ESI-TOF, M+H) *m/z*: calcd. for ($C_{12}H_{24}NO_5Se$): 342.0820, found: 342.0816.

N-Acetylmannosamine kinase and *N*-acetylglucosamine kinase expression and purification

Human *N*-acetylmannosamine kinase (MNK) was expressed and purified using an established method.[1] Therefore, his-tagged MNK was expressed in BL21-CodonPlus (DE3)-RIL *Escherichia coli* (Stragene). Purification was performed by Ni-NTA affinity chromatography, and subsequently size exclusion chromatography on a Superdex™ HighLoad 16/600 column (GE Healthcare).
GST-Tagged human *N*-acetylglucosamine kinase (GNK) was expressed in BL21-CodonPlus(DE3)-RIL Escherichia coli (Stragene) as described.[2] Purification was performed by glutathione affinity chromatography, followed by size exclusion chromatography on a Superdex™ HighLoad 16/600 column (GE Healthcare).
To evaluate their purity, protein samples were mixed with SDS-PAGE sample buffer, denatured at 90 °C for 1 min and separated by a 10% SDS-PAGE gel. Proteins were visualized with Coomassie G-250 staining solution (Biorad), and PageRuler Prestained Plus (Biorad) was used as a marker (Figure S1).

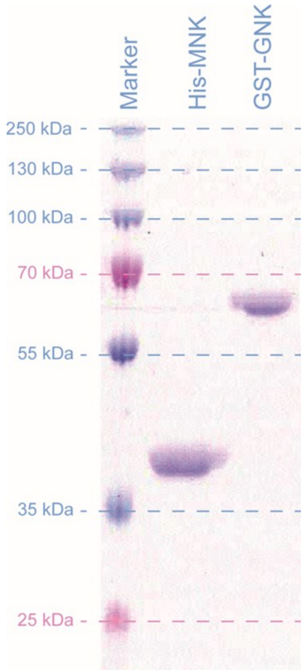

Figure S1. SDS-PAGE of His-tagged MNK (His-MNK) and GST-tagged GNK (GST-GNK) after affinity chromatography and size exclusion chromatography. Apparent molecular masses given by the marker are indicated on the left side.

[1] J. Martinez, L. D. Nguyen, S. Hinderlich, R. Zimmer, E. Tauberger, W. Reutter, W. Saenger, H. Fan and S. Moniot, *J. Biol. Chem.*, 2012, **287**, 13656.
[2] W.A. Weihofen, M. Berger, H. Chen, W. Saenger, and S. Hinderlich, *J. Mol. Biol.*, 2006, **364**, 388.

Enzyme activity and inhibition studies using *N*-acetylmannosamine kinase, *N*-acetylglucosamine kinase and yeast hexokinase

*Evaluation MNK activity in presence of the compounds **11, 12, 14b, 16b** and **18b***

The effects of compounds **11**, **12**, **14b**, **16b**, or **18b** on MNK enzyme activity were evaluated using a coupled optical assay. 55 µl reaction mixture contained the following buffers and reagents: 39.5 µl buffer A (65 mM $MgCl_2$, 200 mM Tris-HCl, pH 8.1), 5 µl ATP (50 mM), 3.5 µl NADH (30 mM), 2 µl phosphoenolpyruvate (100 mM), 2 U pyruvate-kinase, 2 U lactate-dehydrogenase, 5 µl ManNAc of varying concentrations. Subsequently, 10 µl of the compounds **11**, **12**, **14b**, **16b** and **18b** were added in varying concentrations. The reaction was initiated by adding 0.6 µg MNK in 35 µL buffer B (10 mM Tris-HCl, 150 mM NaCl, pH 8.0). Samples were incubated at 37°C. Spectrophotometric measurements of NADH concentration was performed at 340 nm. Results obtained were normalized to blanks, consisting of 55 µl reaction mixture, 10 µl H_2O and 35 µl buffer B without MNK.

*GNK and hexokinase inhibition assays using compound **14b***

To evaluate the specificity of the inhibitor **14b** towards other sugar kinases, we performed further inhibition experiments using human GNK and hexokinase (HK) from yeast (Sigma, H-5000). 39.5 µl Buffer A (see above), 5 µl ATP (50 mM), 3.5 µl NADH (30 mM), 2 µl phosphoenolpyruvate (100 mM), 2 U pyruvate-kinase, 2 U lactate-dehydrogenase, 5 µl GlcNAc (7.5 mM, for GNK experiments), or 5 µl ManNAc (7.5 mM, for GNK experiments), or glucose (5 mM, for HK experiments), and 10 µl **14b** (varying concentrations) were mixed with either 1 µg GNK or 0.2 µg HK in 35 µL buffer B. The decrease of NADH was measured in a spectrophotometer as described above. The IC_{50} of **14b** for GNK activity was 1.7 mM (± 0.7 mM), and > 5 mM for HK activity, respectively (Figure S2).

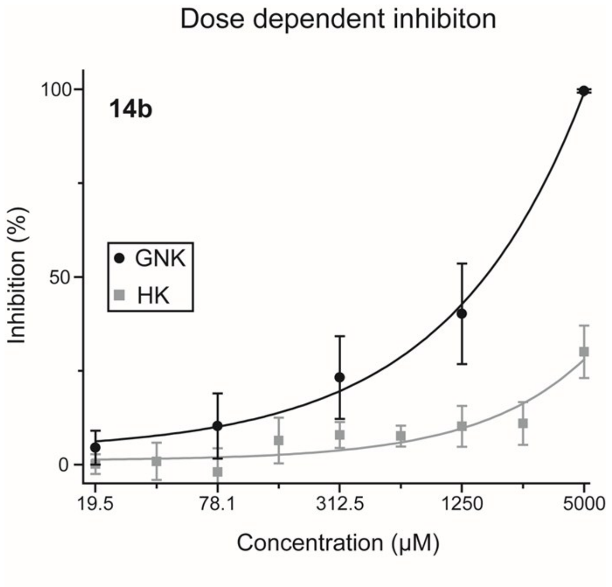

Figure S2. Inhibition of GNK and HK activity by **14b**. Values are means ± SEM of triplicates

Evaluation of the influence of 14b on UDP-GlcNAc-2-epimerase activity

To study, if diselenide **14b** interferes with the activity of the GNE domain of the bifunctional enzyme, we used enzymes from mouse liver homogenates. Herein, 5 ml of buffer D (10 mM sodium phosphate, pH 7.1, 1 mM EDTA, 1 mM DTT and cOmplete Protease inhibitor cocktail (Roche) were added to shock-frozen mouse liver (≈ 1 g). The sample was disrupted using an Ultra-Turrax T25 (IKA Labortechnik) twice for 10 s, followed by centrifugation at 35.000 x g for 20 min. The cytosolic supernatant was used for a colorimetric GNE activity assay as reported.[3] In brief, a total volume of 200 µL contained 45 mM sodium phosphate, pH 7.5, 10 mM $MgCl_2$, 1 mM UDP-GlcNAc and 100 µL of mouse liver cytosol. The assay was incubated for 45 min at 37 °C and stopped by incubation at 95 °C for 3 min. After a short centrifugation to precipitate insoluble compounds 150 µL of the supernatant were mixed with 30 µL of 0.8 M borate, pH 9.1, and boiled for 10 min. Then, 800 µL of 1% (w/v) 4-dimethylamino benzaldehyde and 1.25% (v/v) 10 M HCl in acetic acid were added. The sample was incubated at 37 °C for 20 min and the absorbance was measured at 578 nm. The IC50 of **14b** for inhibiting GNE activity was determined to be 182 ± 13 µM (Figure S3).

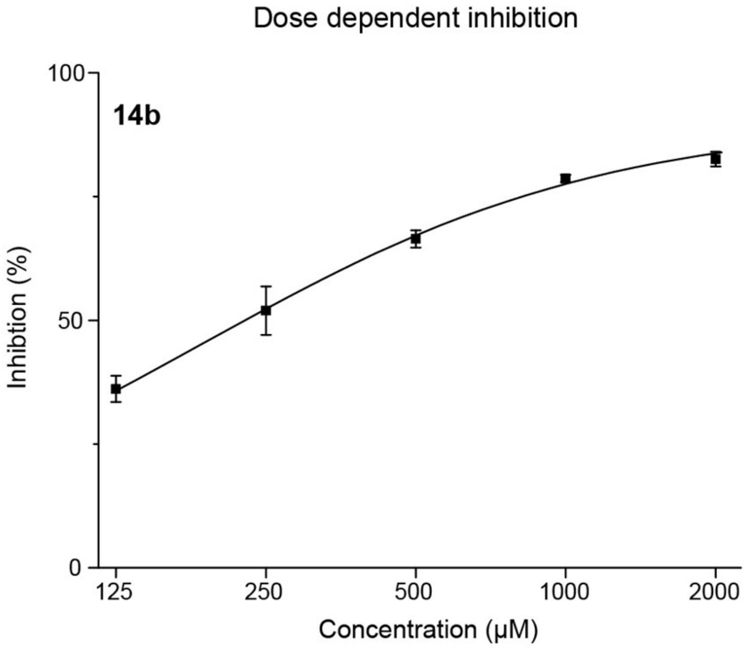

Figure S3. Inhibition of UDP-GlcNAc-2 epimerase activity by **14b**. Values are means ± SEM of four experiments.

[3] W. Reutter, S. Hinderlich and W. Kemmner "UDP-GlcNAc-2-epimerase/ManNAc kinase (GNE), section XV, pp 1511-1522 in N. Taniguchi, K. Honke, M. Fukuda, H. Narimatsu, Y. Yamaguchi and T. Angata eds., *Handbook of Glycosyltransferases and Related Genes*, 2nd edn., Springer Japan, Japan, 2014.

Cytotoxicity studies

The cytotoxicity of compounds **14a** and **16a** in Jurkat cells was evaluated utilizing the AlamarBlue© assay (AbD Serotec). Approximately 20,000 Jurkat cells (ATCC) were cultured for 72 h in RPMI 1640 medium (10 % FBS, 2 mM L-glutamine) containing different concentrations of the peracetylated compounds **14a** and **16b**. 10 µl AlamarBlue© solution was added, and the cells were incubated for another 4 h. Samples were analyzed in a spectrophotometer at wavelengths of 570 and 620 nm. Experiments were performed in triplicate and normalized to untreated cells (100 % viability) as well as blanks (0 % viability) consisting of cell culture medium without cells (Figure S4).

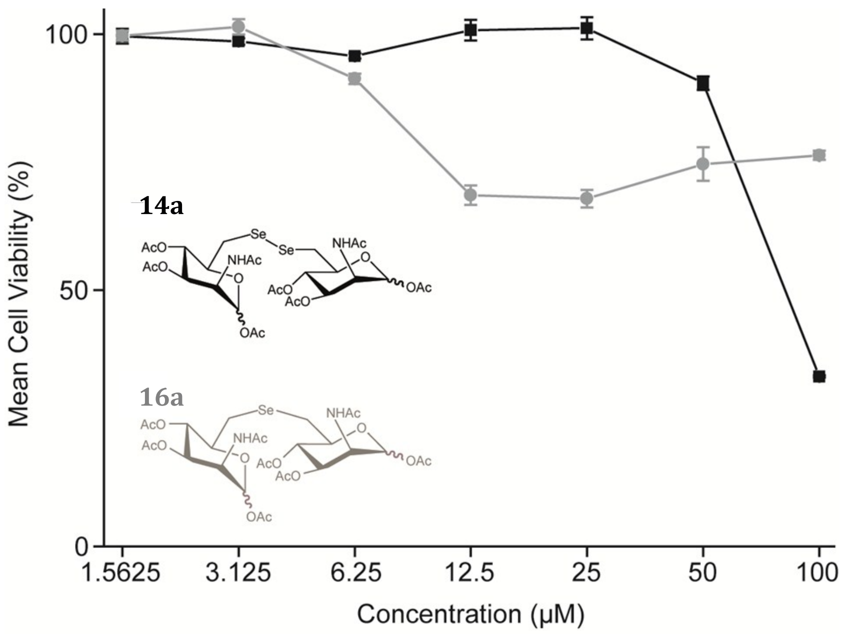

Figure S4. Evaluation of cytotoxicity effects of diselenide **14a** and selenide **16a** in Jurkat cells. Data shown represent the mean values and S.E.M obtained in a triplicate.

Determination of cell surface sialylation

Approximately 20,000 Jurkat cells (ATCC) were cultured for 72 h in 100 µl RPMI 1640 medium with 10 % FBS and 2 mM L-glutamine, containing **14b** or **16b** in varying concentrations. After incubation cells were washed three times with PBS containing 0.5 % bovine serum albumin (PBS + 0.5 % BSA) and labeled for 1 h at 4°C with FITC-conjugated *Polyporus squamosus* lectin (PSL, 0.1 µg/ml). Subsequently, cells were washed three times with PBS + 0.5 % BSA and analyzed on a FACSCanto II flowcytomer (BD Biosciences). Obtained data was normalized to untreated cells and cells treated for 60 min with 0.2 U/ml sialidase from *Clostridium perfringens* (Sigma Aldrich). In order to minimize errors caused by dead cells, only intact cells have been included into the data evaluation, whereas dead cells, small debris and large clumps have been excluded using a probe of untreated cells stained with propidium iodide. Experiments were performed in a triplicate

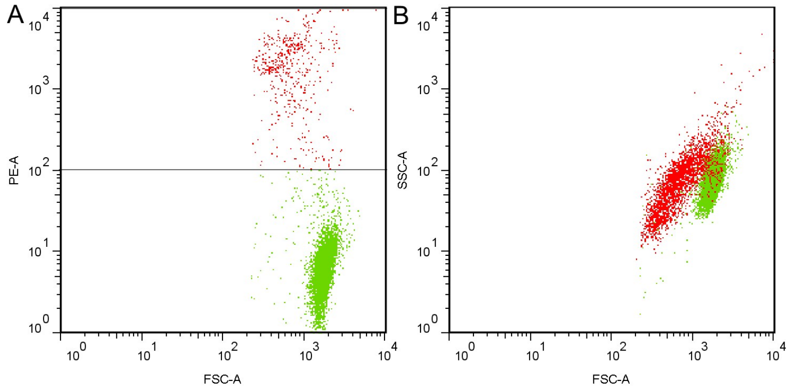

Figure S5. Differentiation between live and dead cells in flow cytometry experiments. In order to exclude dead cells from analysis, a probe of untreated Jurkat cells was stained with propidium iodide (PI) and injected into the flow cytometer. Dot plot (A) depicts forward scattering of light against PE fluorescence (FSC/PE). Red dots represent highly fluorescent dead cells, whereas green dots represent cells that are low fluorescent and therefore considered as being alive. (B) Shows the distribution of PI stained live and dead cells with forward- against side scattering of light (FSC/SSC). To analyze the experiments with FITC-conjugated *Polyporus squamosus* lectin, gates have been used that exclude most of the dead cell population based on this experiment with PI stained untreated cells (see **figure S6**).

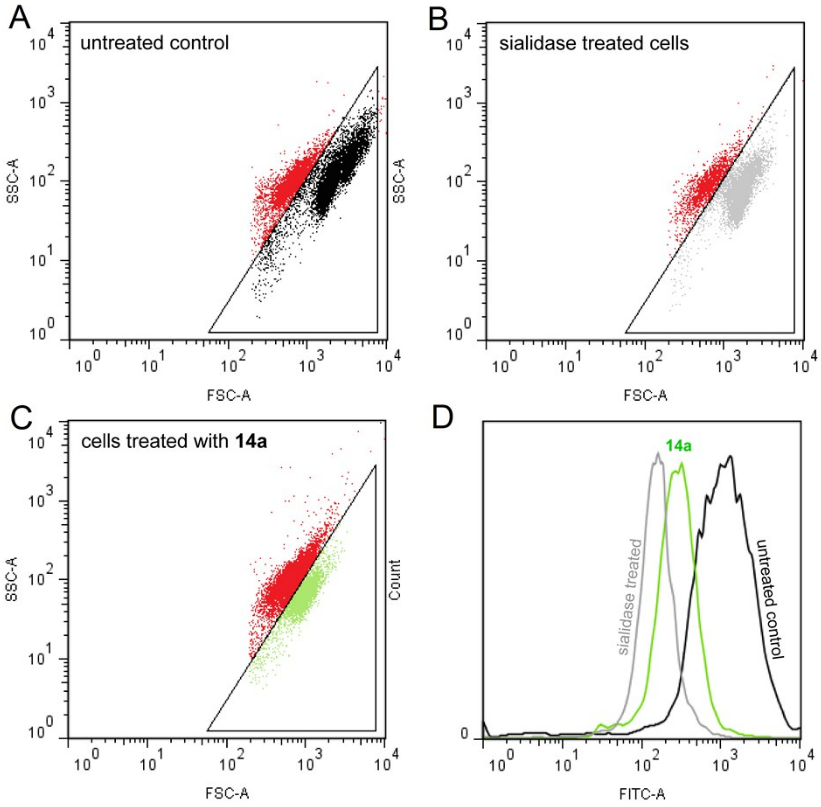

Figure S6. Flow cytometry analysis of Jurkat cells stained with FITC-conjugated *Polyporus squamosus* lectin (PSL). (A) untreated cells, (B) cells treated with sialidase, and (C) cells treated with 50 μM **14a** (C). Triangular gates have been used

to exclude dead cells, small debris and large clumps (red dots, see **figure S5**). Representative histogram (D) shows that cells treated with 50 μM **14a** expose less fluorescence than untreated control cells, which indicates lower levels of cell surface sialylation in these inhibitor treated cells.

NMR spectra

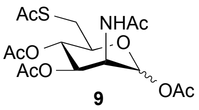
AcS
NHAc
O
AcO
AcO
OAc
9

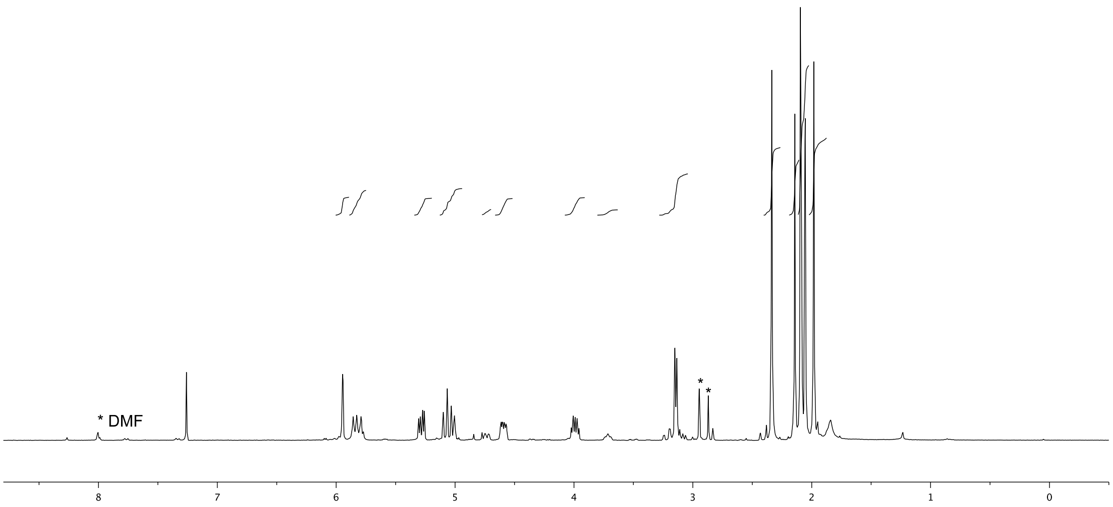
* DMF
*
*
8
7
6
5
4
3
2
1
0

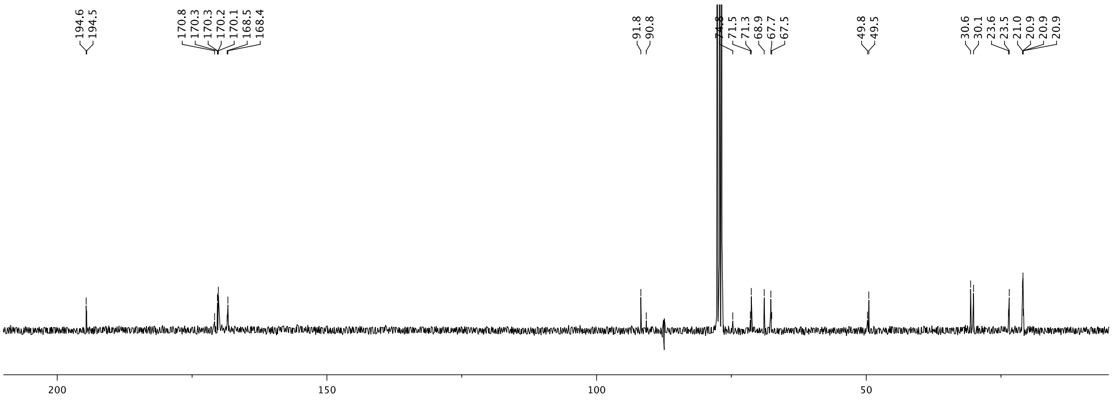
194.6
194.5
170.8
170.3
170.3
170.2
170.1
168.5
168.4
91.8
90.8
74.8
71.5
71.3
68.9
67.7
67.5
49.8
49.5
30.6
30.1
23.6
23.5
21.0
20.9
20.9
20.9
200
150
100
50

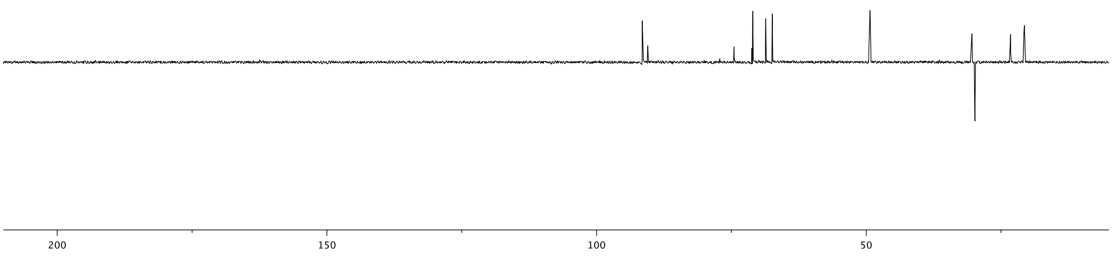
200
150
100
50

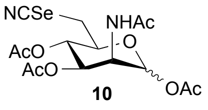
NCSe
NHAc
O
AcO
AcO
OAc
10

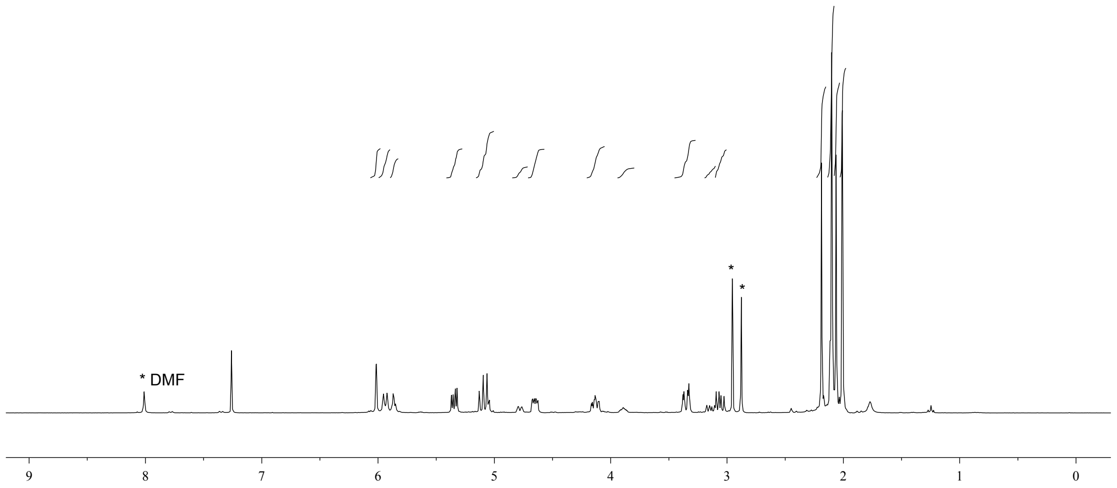
* DMF
*
*
9
8
7
6
5
4
3
2
1
0

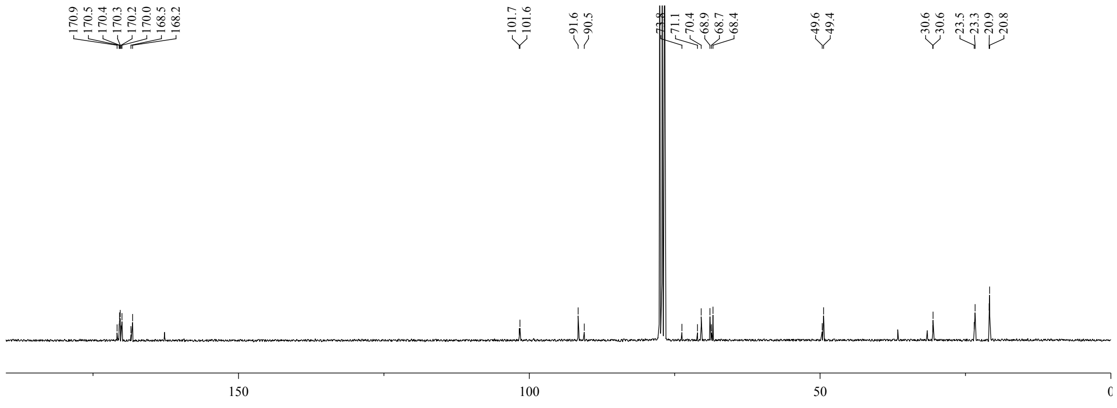
170.9
170.5
170.4
170.3
170.2
170.0
168.5
168.2
101.7
101.6
91.6
90.5
71.1
70.4
68.9
68.7
68.4
49.6
49.4
30.6
30.6
23.5
23.3
20.9
20.8
150
100
50

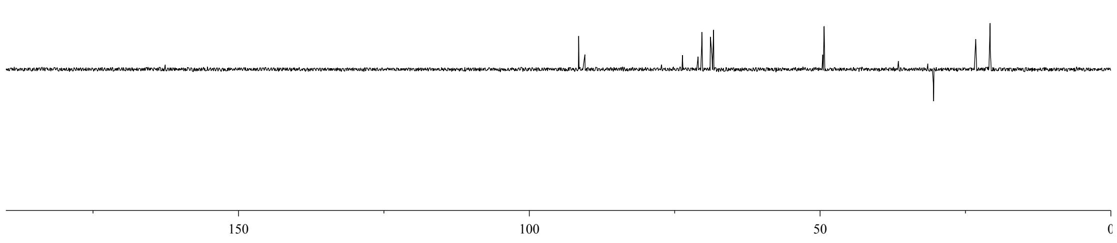
150
100
50

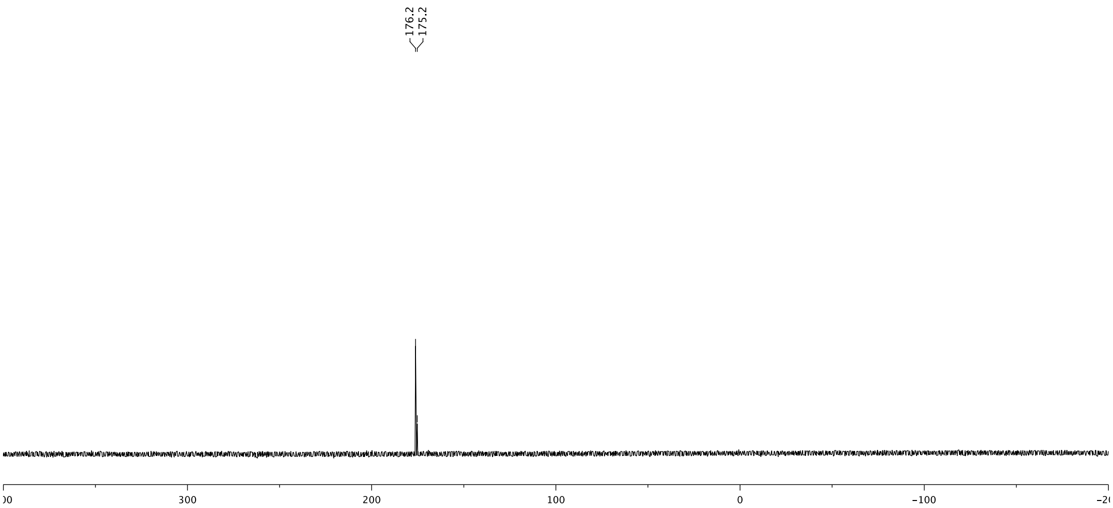

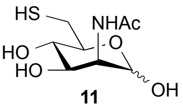

11

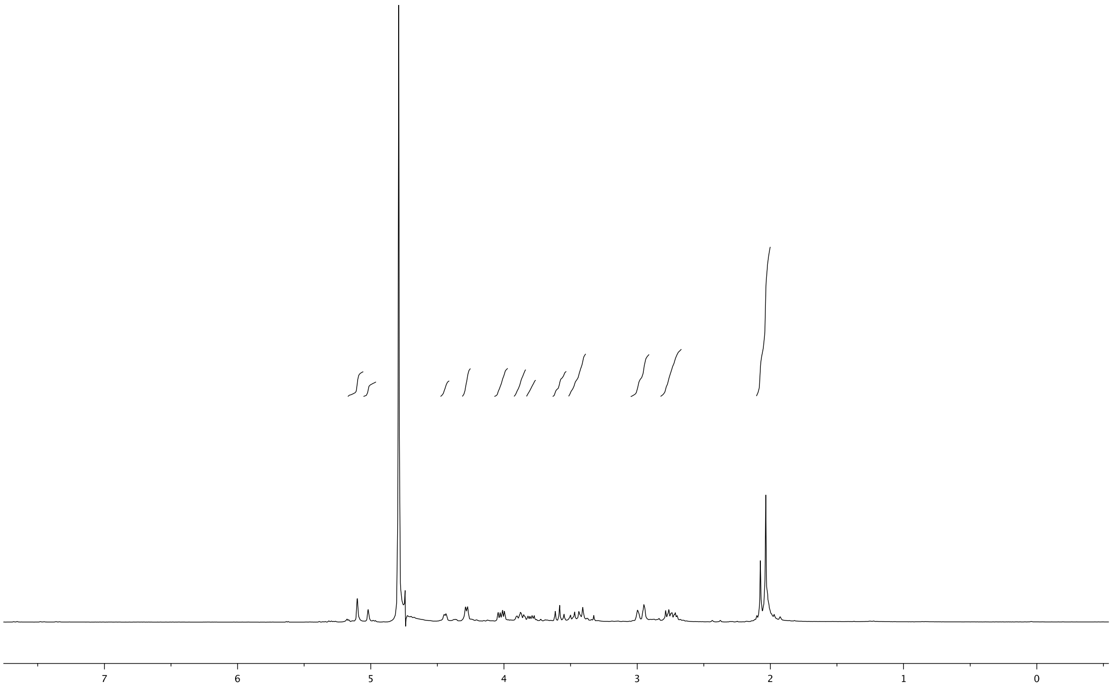

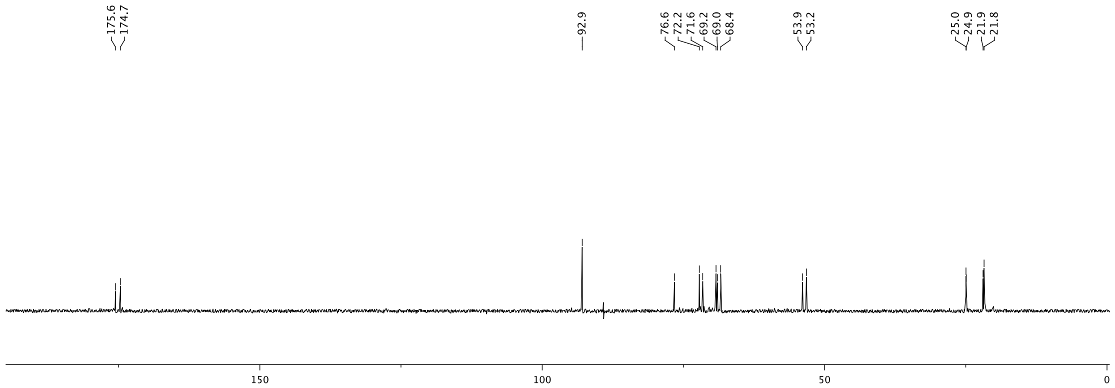
175.6
174.7
92.9
76.6
72.2
71.6
69.2
69.0
68.4
53.9
53.2
25.0
24.9
21.9
21.8
150
100
50
0

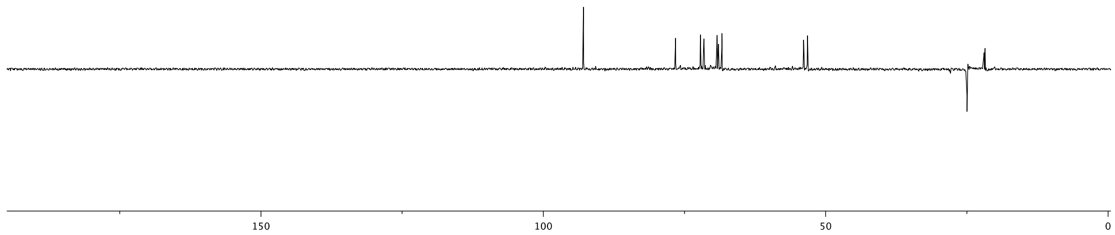
150
100
50
0

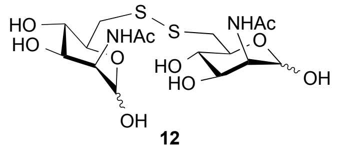
HO
HO
NHAc
O
OH
S
S
NHAc
O
HO
HO
OH

12

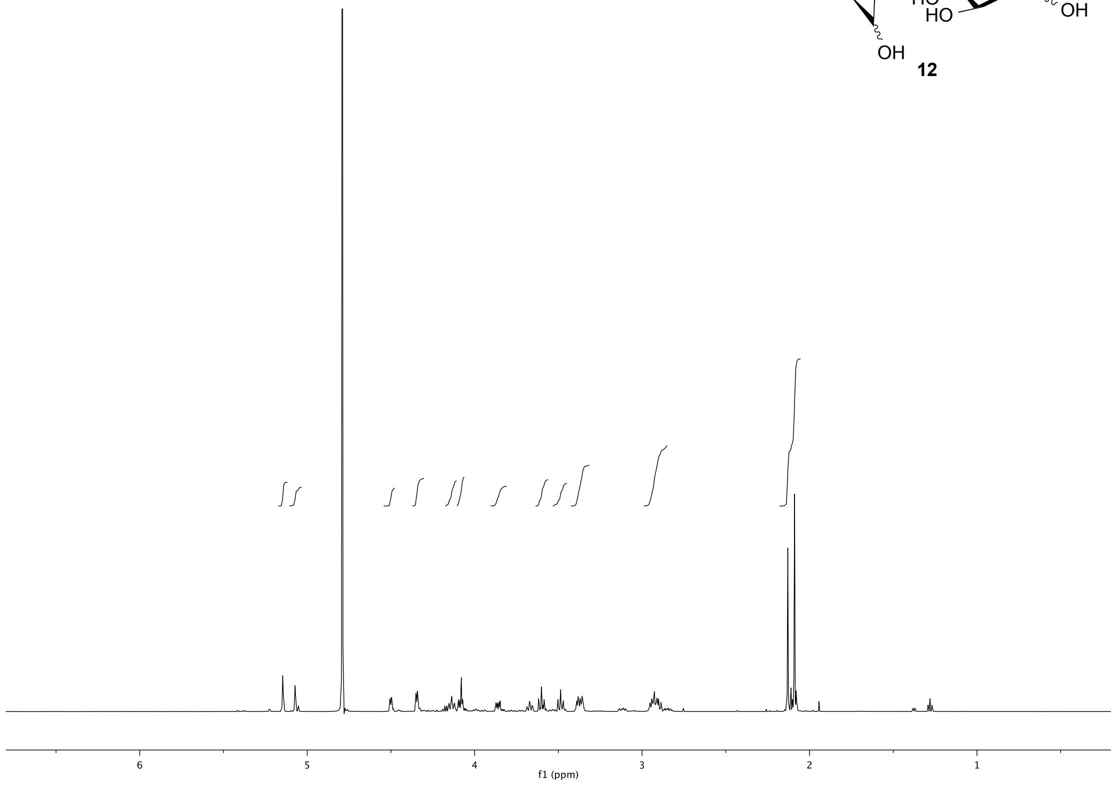
6
5
4
3
2
1
f1 (ppm)

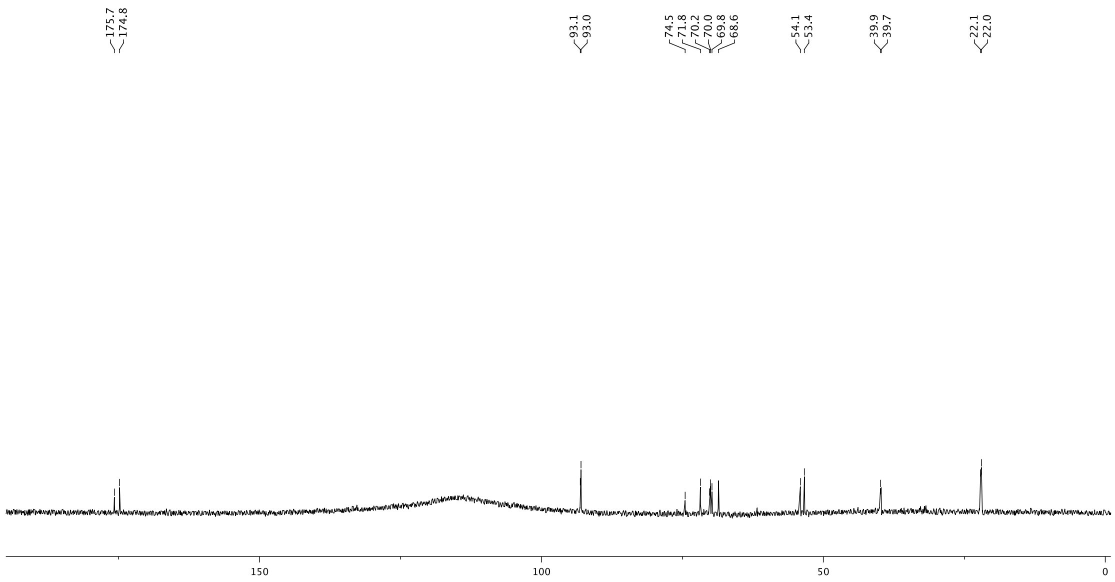
175.7
174.8
93.1
93.0
74.5
71.8
70.2
70.0
69.8
68.6
54.1
53.4
39.9
39.7
22.1
22.0
150
100
50
0

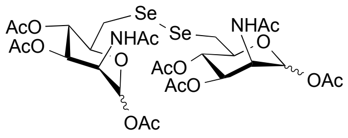
AcO
AcO
Se
Se
NHAc
NHAc
O
O
AcO
AcO
OAc
OAc

14a

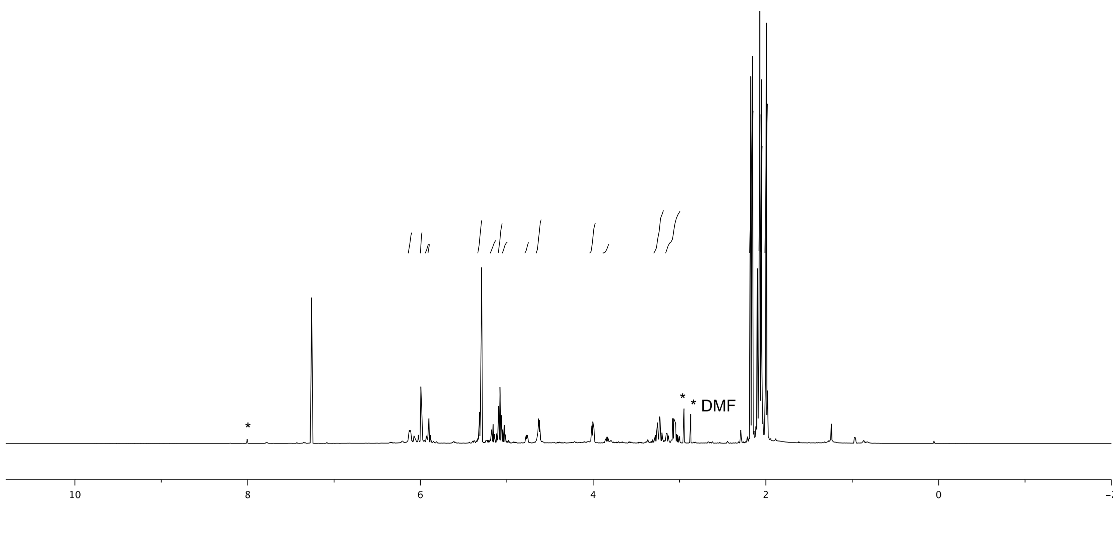
* DMF
10
8
6
4
2
0

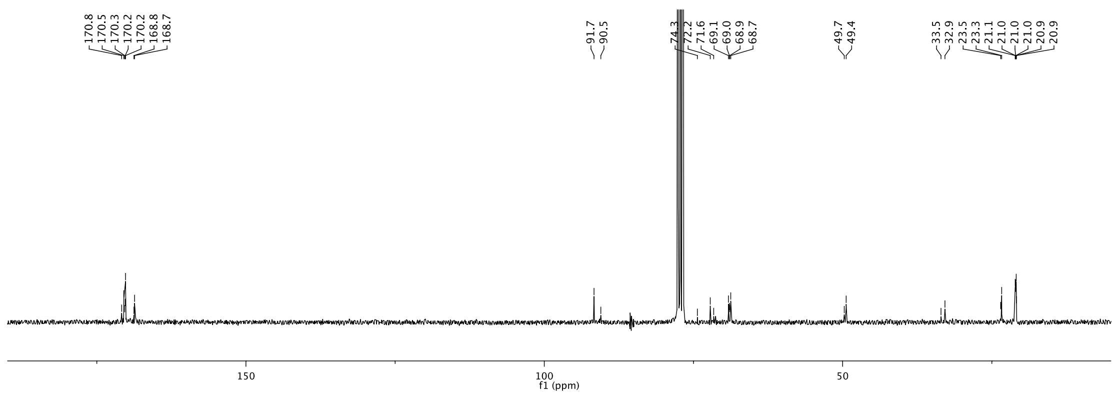
170.8
170.5
170.3
170.2
170.2
168.8
168.7
91.7
90.5
74.3
72.2
71.6
69.1
69.0
68.9
68.7
49.7
49.4
33.5
32.9
23.5
23.3
21.1
21.0
21.0
21.0
20.9
20.9
150
100
50
f1 (ppm)

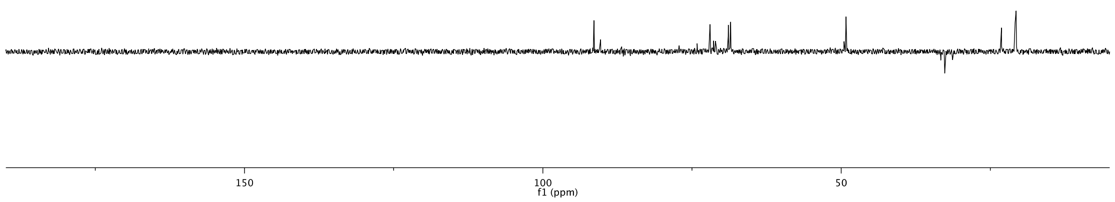
150
100
50
f1 (ppm)

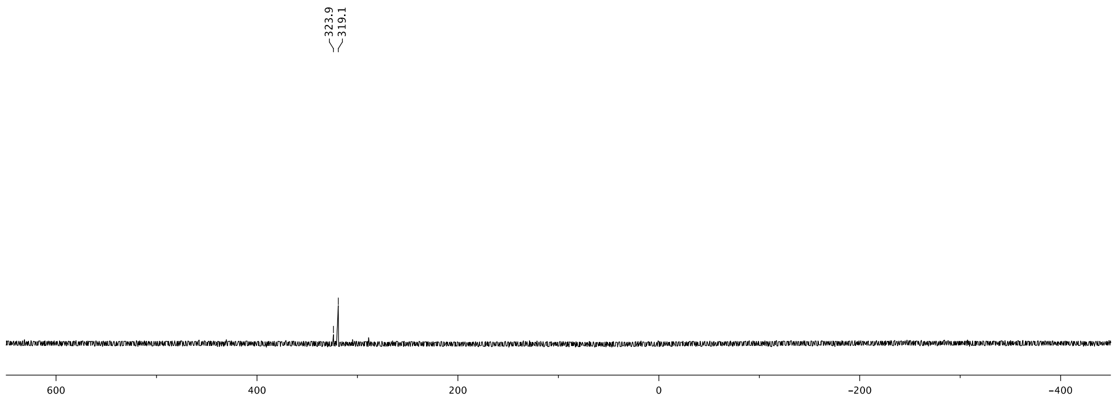
323.9
319.1
600
400
200
0
−200
−400

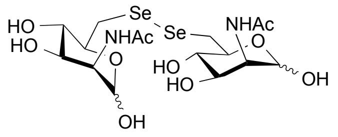
Se
Se
NHAc
NHAc
HO
HO
HO
HO
O
O
OH
OH

14b

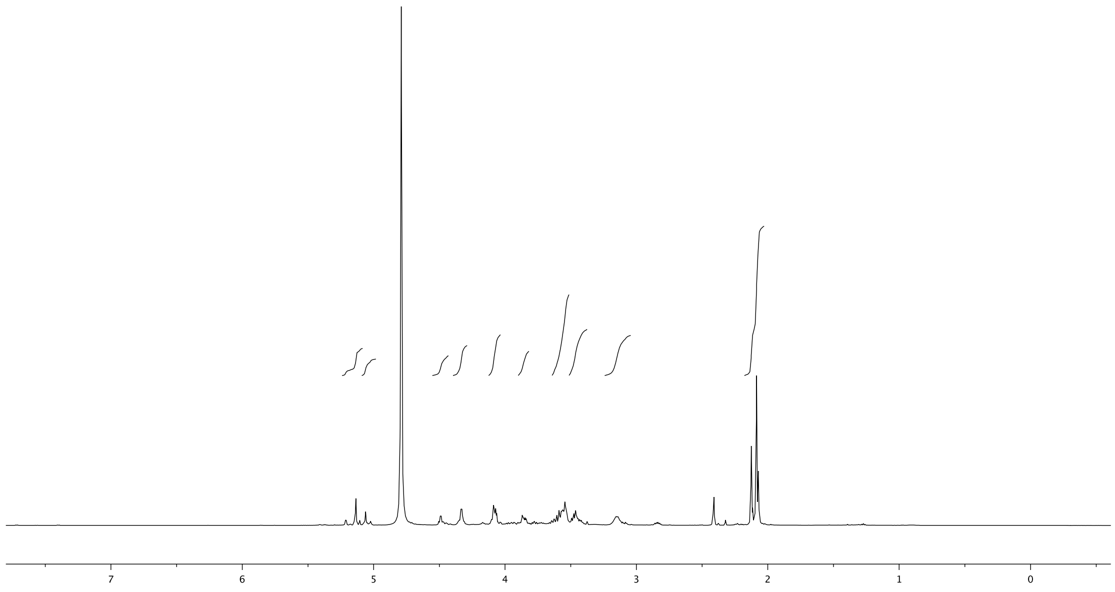
7
6
5
4
3
2
1
0

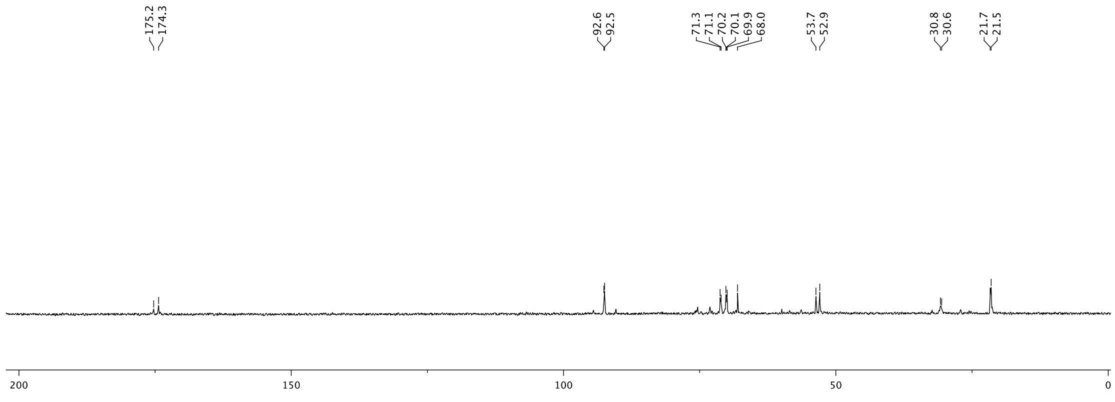
175.2
174.3
92.6
92.5
71.3
71.1
70.2
70.1
69.9
68.0
53.7
52.9
30.8
30.6
21.7
21.5
200
150
100
50
0

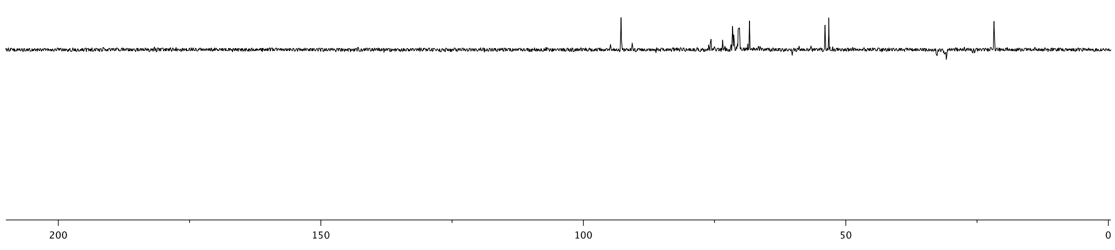
200
150
100
50
0

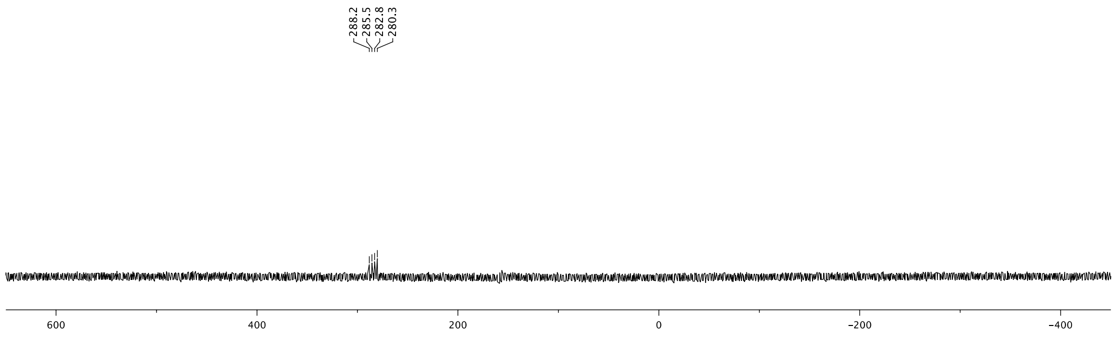
288.2
285.5
282.8
280.3
600
400
200
0
−200
−400

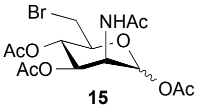
Br
NHAc
O
AcO
AcO
OAc

15

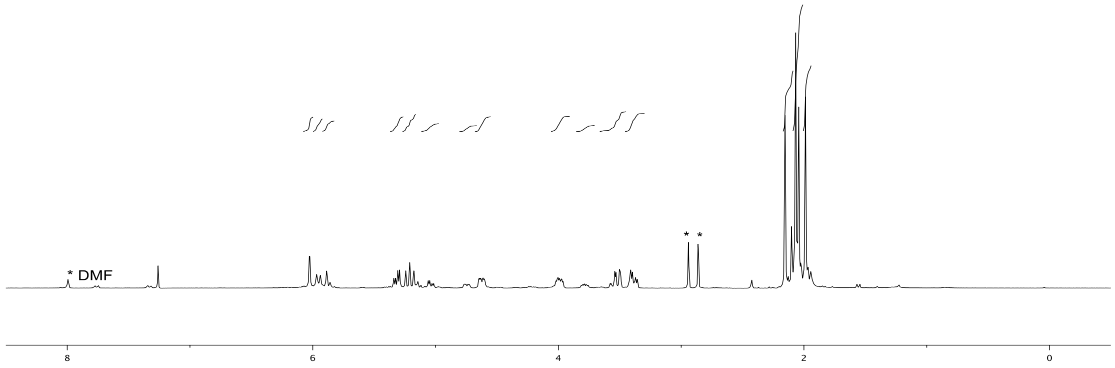
* DMF
8
6
4
2
0

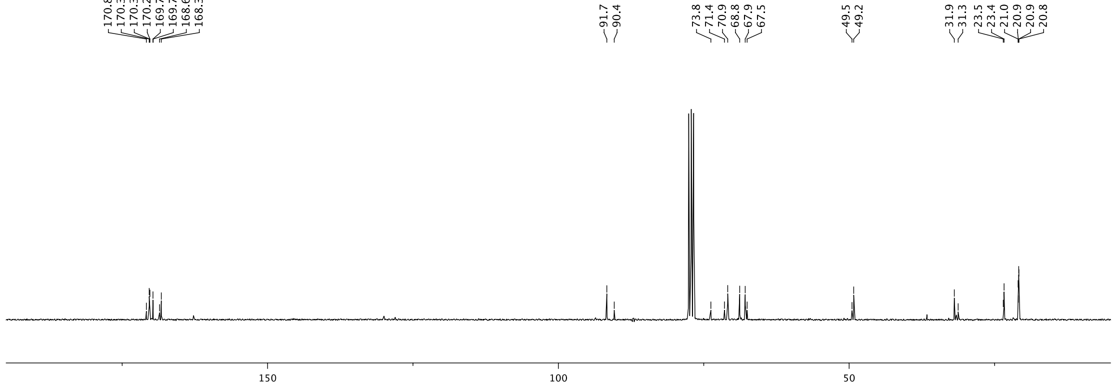
170.8
170.3
170.3
170.2
169.7
169.7
168.6
168.3
91.7
90.4
73.8
71.4
70.9
68.8
67.9
67.5
49.5
49.2
31.9
31.3
23.5
23.4
21.0
20.9
20.9
20.8
150
100
50

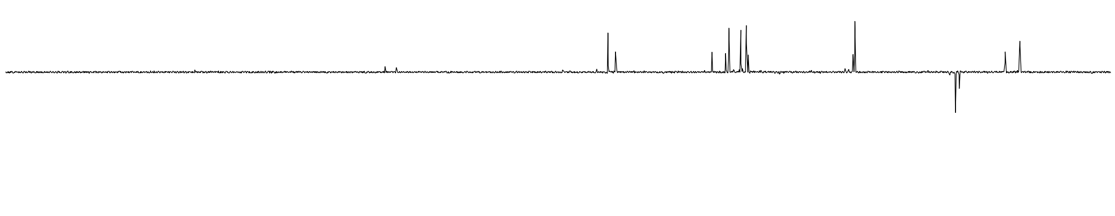
150
100
50

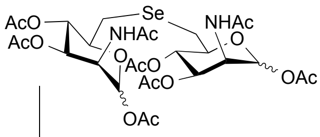
AcO
AcO
Se
NHAc
O
AcO
AcO
NHAc
O
OAc
OAc

16a

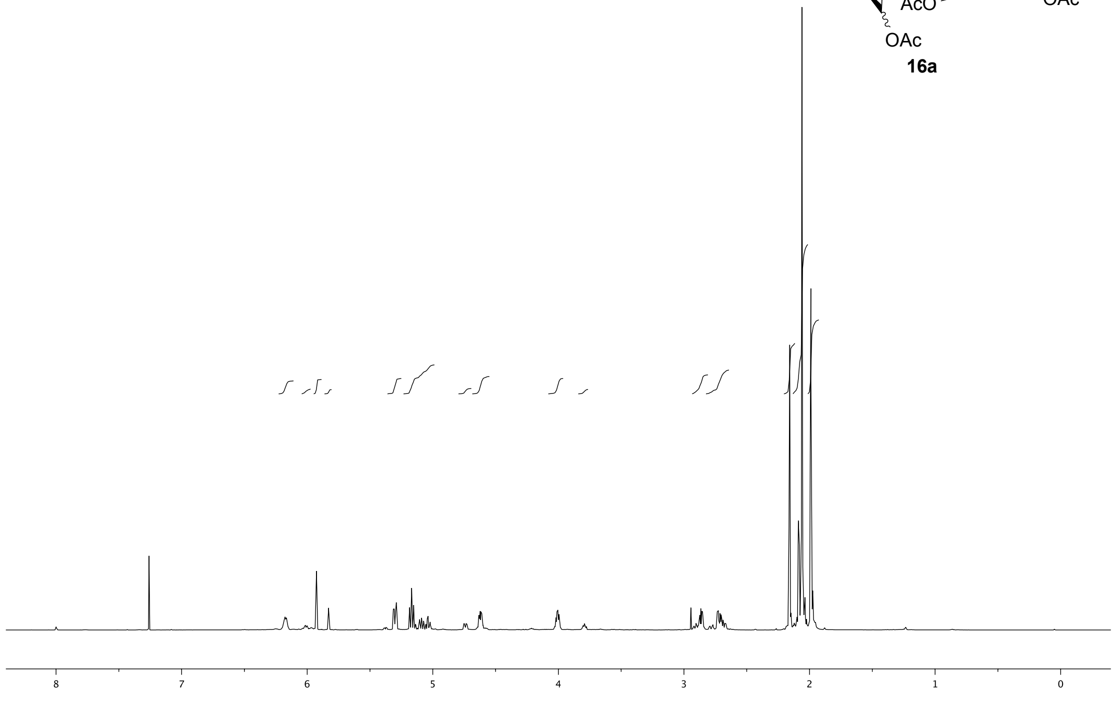
8
7
6
5
4
3
2
1
0

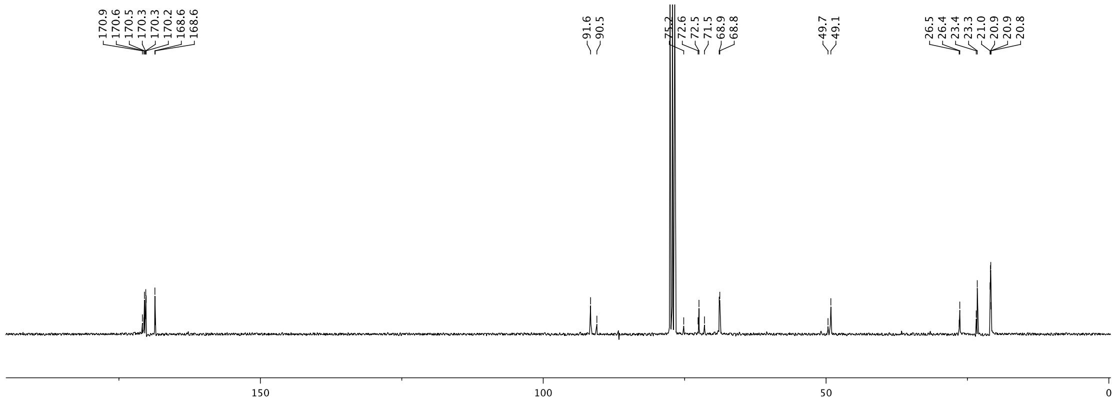
170.9
170.6
170.5
170.3
170.3
170.2
168.6
168.6
91.6
90.5
75.2
72.6
72.5
71.5
68.9
68.8
49.7
49.1
26.5
26.4
23.4
23.3
21.0
20.9
20.9
20.8
150
100
50
0

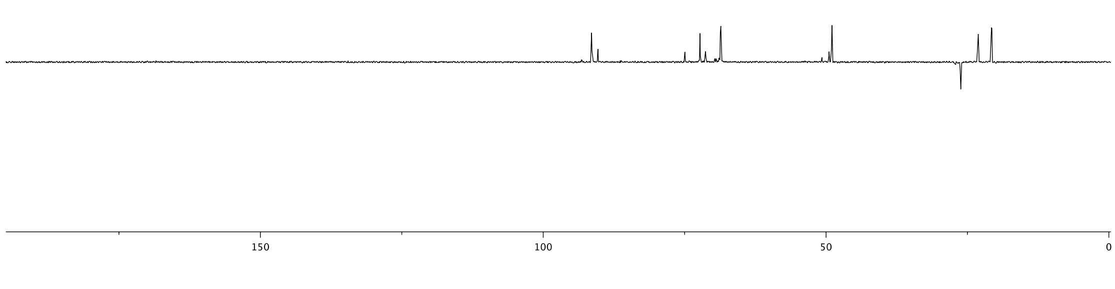
150
100
50
0

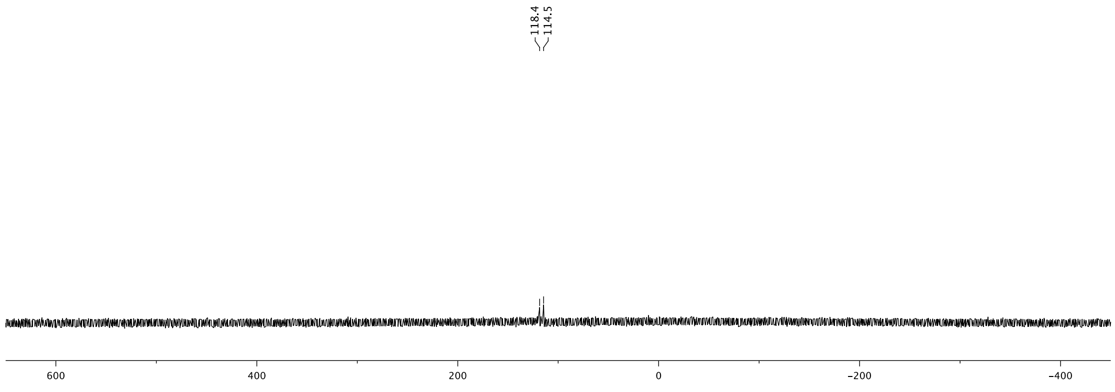

118.4
114.5
600
400
200
0
−200
−400

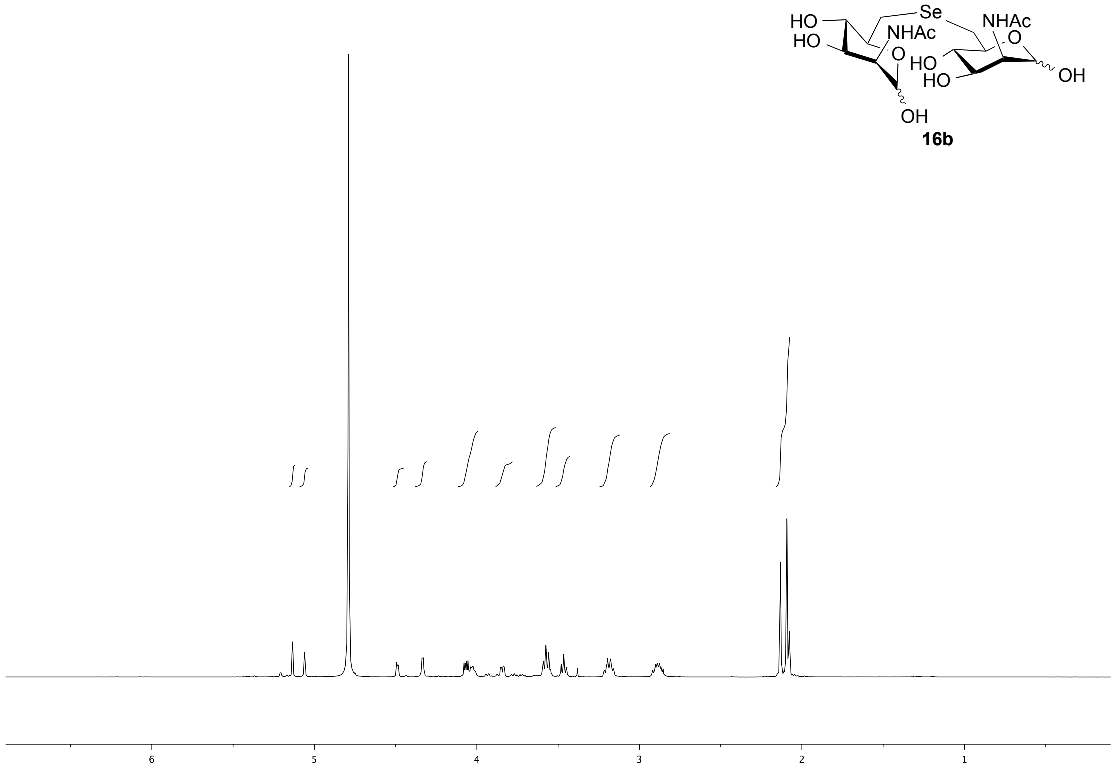

HO
HO
Se
NHAc
O
OH
NHAc
O
HO
HO
OH
16b
6
5
4
3
2
1

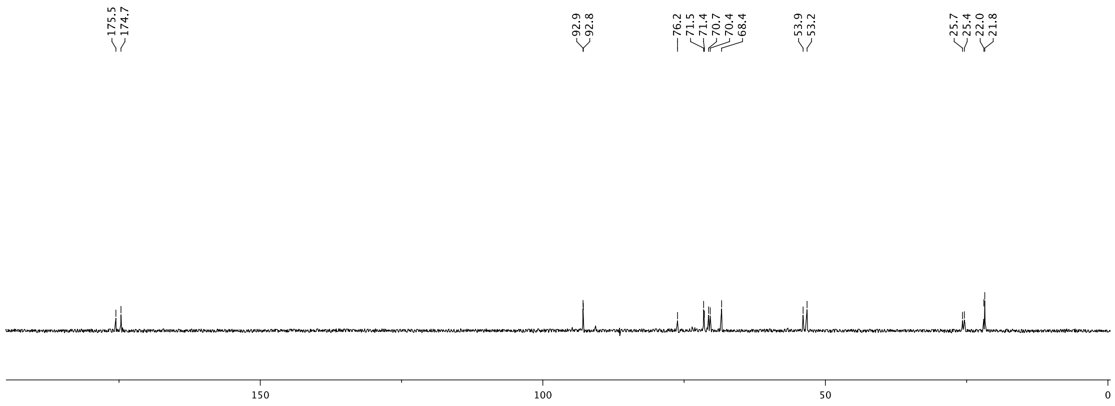
175.5
174.7
92.9
92.8
76.2
71.5
71.4
70.7
70.4
68.4
53.9
53.2
25.7
25.4
22.0
21.8
150
100
50
0

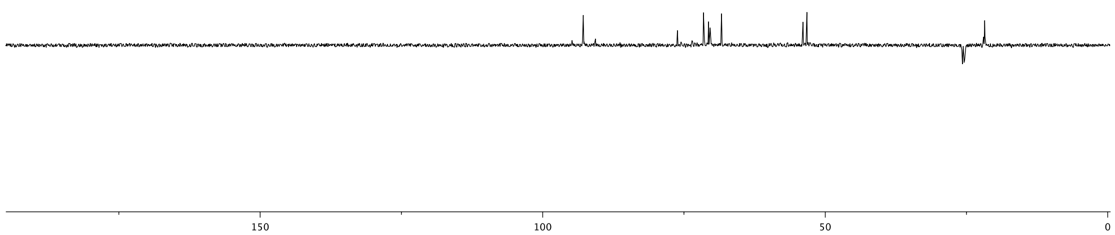
150
100
50
0

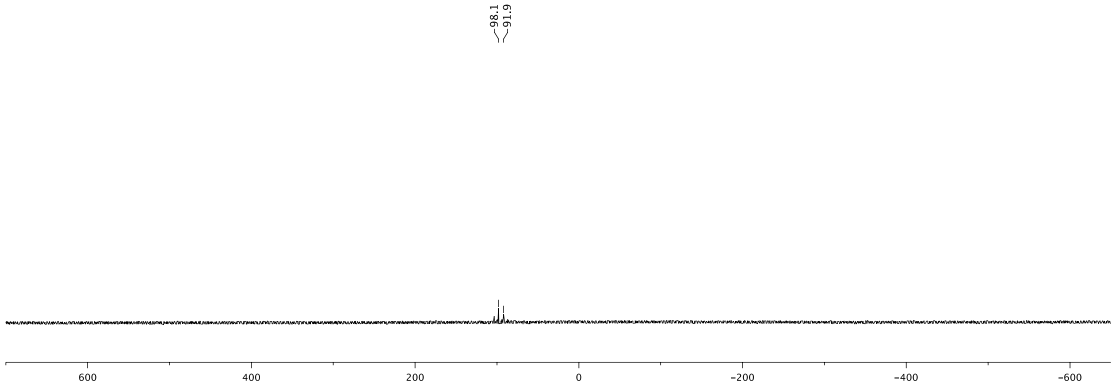
98.1
91.9
600
400
200
0
−200
−400
−600

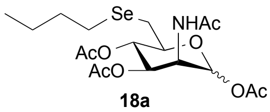
Se
NHAc
O
AcO
AcO
OAc

18a

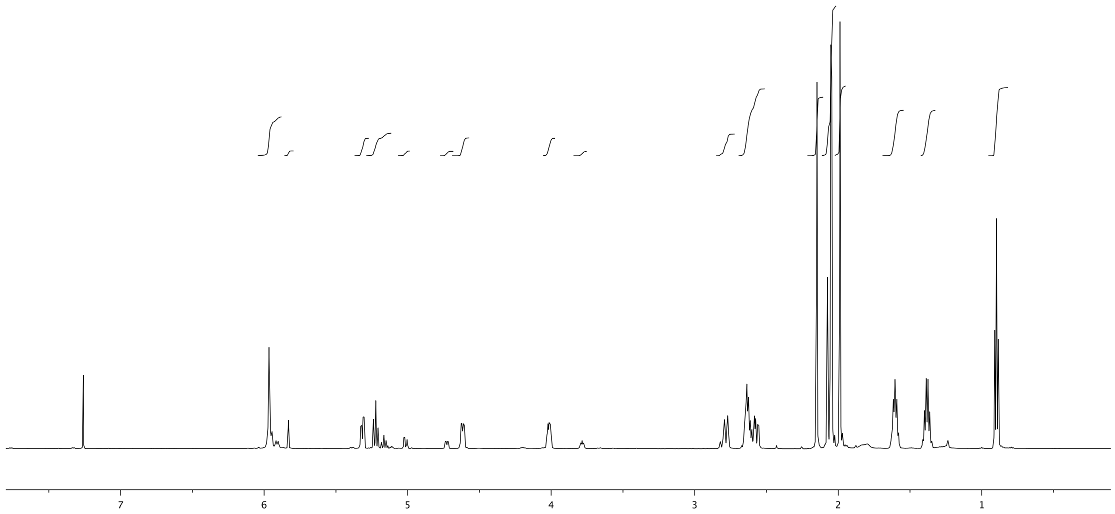
7
6
5
4
3
2
1

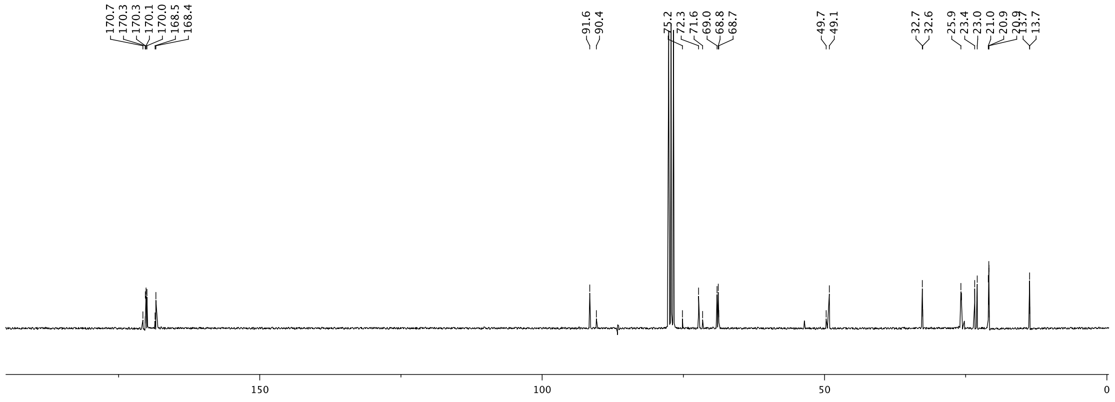
170.7
170.3
170.3
170.1
170.0
168.5
168.4
91.6
90.4
75.2
72.3
71.6
69.0
68.8
68.7
49.7
49.1
32.7
32.6
25.9
23.4
23.0
21.0
20.9
13.7
13.7
150
100
50
0

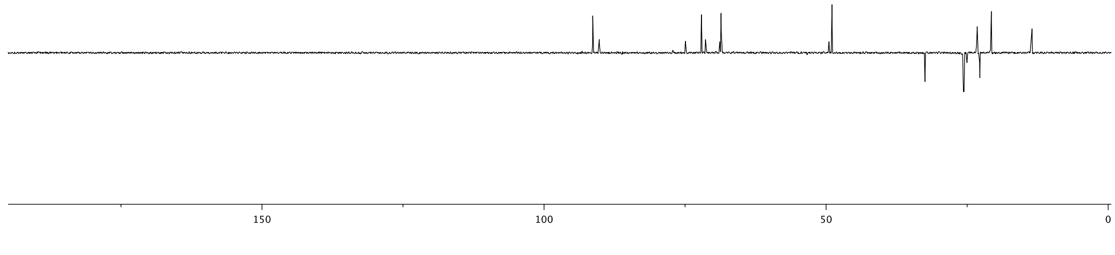
150
100
50
0

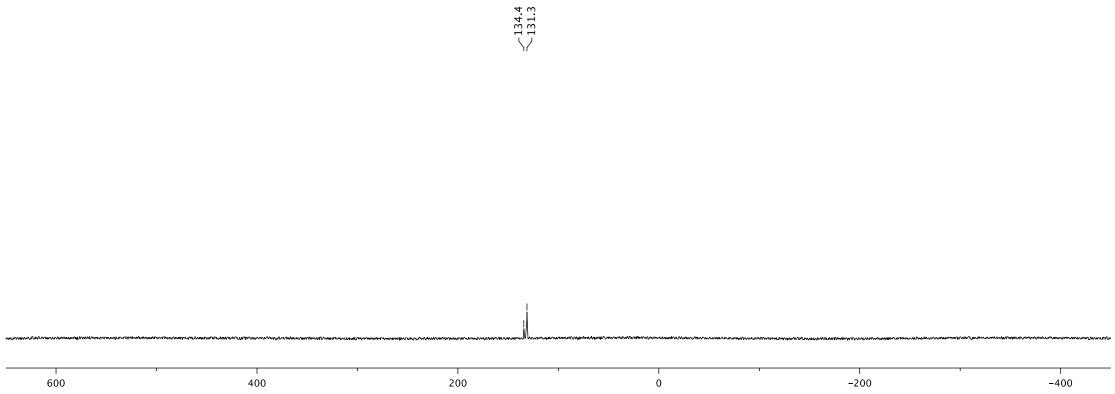

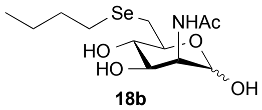

18b

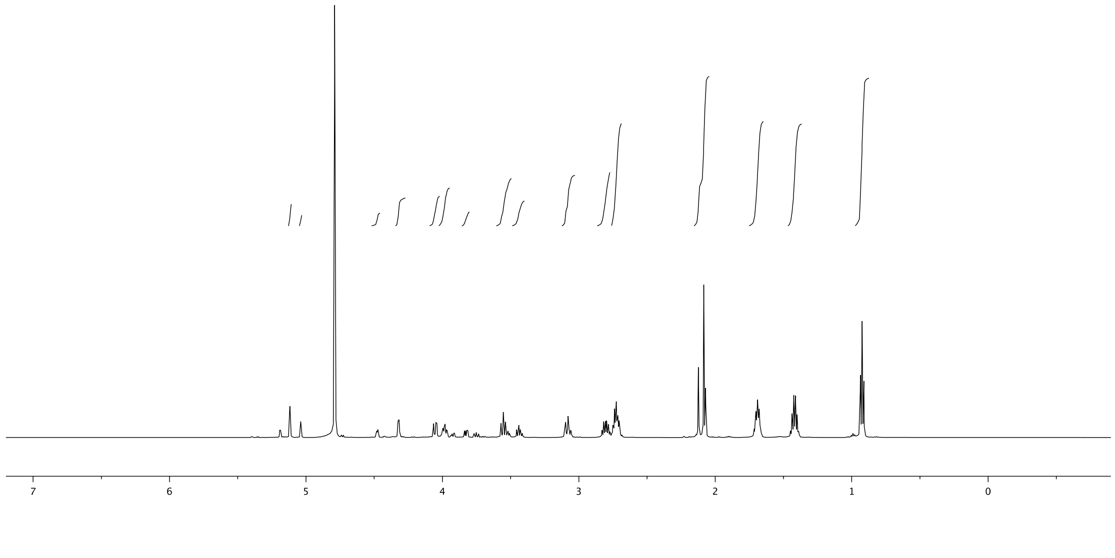

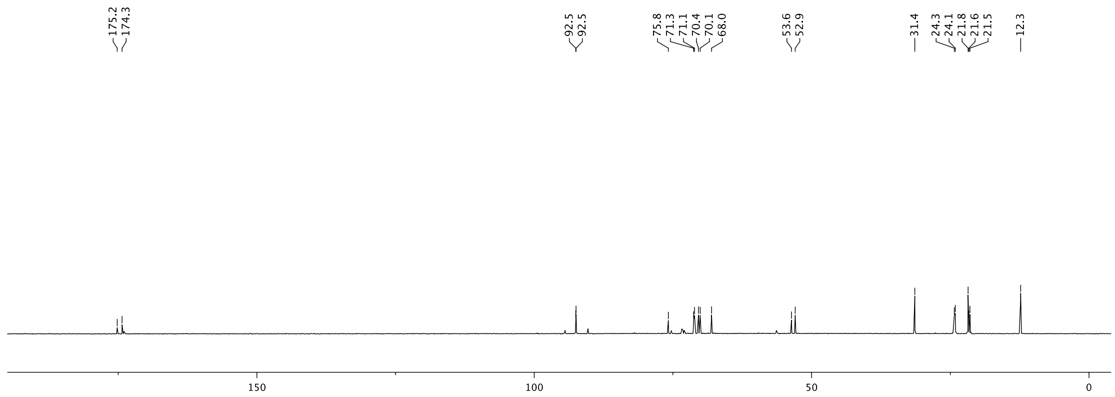
175.2
174.3
92.5
92.5
75.8
71.3
71.1
70.4
70.1
68.0
53.6
52.9
31.4
24.3
24.1
21.8
21.6
21.5
12.3
150
100
50
0

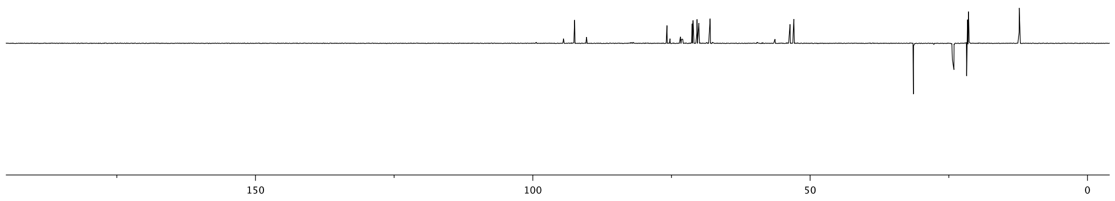
150
100
50
0

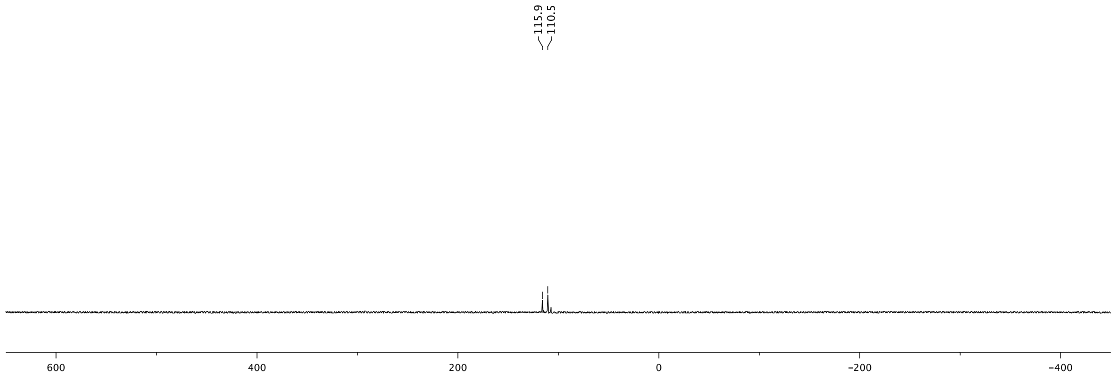
115.9
110.5
600
400
200
0
−200
−400

Evaluation of the stability of the diselenide 14b in presence of dithiothreitol (DTT)

The possible reduction of diselenide bond was monitored by ^{77}Se NMR using a 60 mM solution of diselenide **14b** in aqueous saturated DTT solution at room temperature.

Immediately after the addition of **14b** to the DTT solution:

Diselenide **14b**/selenol (90:10): ^{77}Se NMR (114 MHz, D_2O) δ: 305.0, 299.8, 294.5, 293.0/-48.1, -48.5.

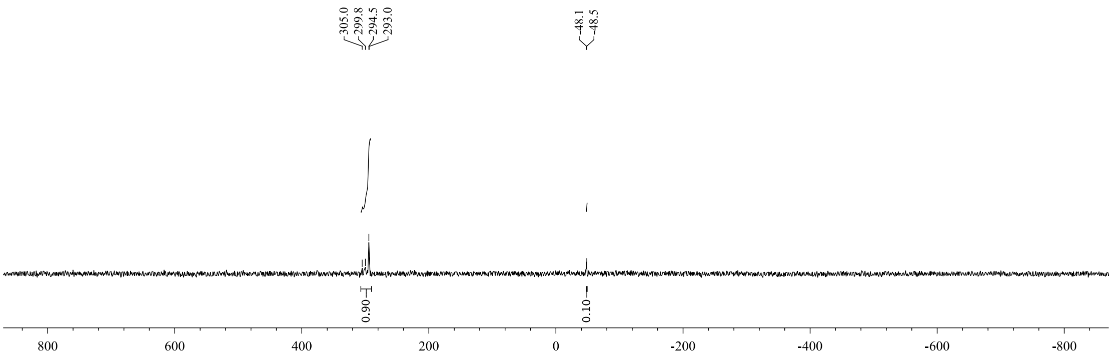

Time period between 15 min and 5 h:

Diselenide **14b**: ^{77}Se NMR (114 MHz, D_2O) δ: 299.6, 298.6, 293.5, 292,2.

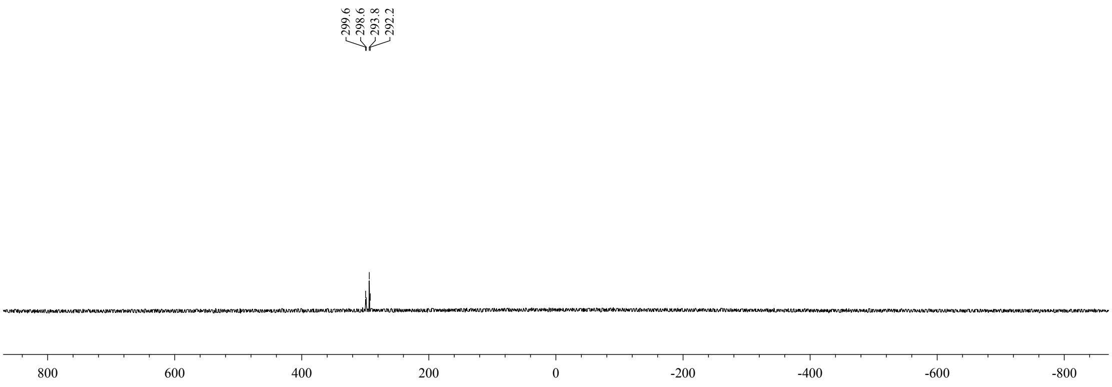

The same experiment was performed using saturated glutathione solution instead of saturated DTT solution. In this case, only diselenide **14b** was detected.
